# Meirols A–C: Bioactive Catecholic Compounds from the Marine-Derived Fungus *Meira* sp. 1210CH-42

**DOI:** 10.3390/md22020087

**Published:** 2024-02-14

**Authors:** Min Ah Lee, Jong Soon Kang, Jeong-Wook Yang, Hwa-Sun Lee, Chang-Su Heo, Sun Joo Park, Hee Jae Shin

**Affiliations:** 1 Marine Natural Products Chemistry Laboratory, Korea Institute of Ocean Science and Technology, 385 Haeyang-ro, Yeongdo-gu, Busan 49111, Republic of Korea; minah@kiost.ac.kr (M.A.L.); hwasunlee@kiost.ac.kr (H.-S.L.); science30@kiost.ac.kr (C.-S.H.); 2 Department of Chemistry, Pukyong National University, 45 Yongso-ro, Nam-gu, Busan 48513, Republic of Korea; parksj@pknu.ac.kr; 3 Laboratory Animal Resource Center, Korea Research Institute of Bioscience and Biotechnology, 30 Yeongudanji-ro, Cheongwon-gu, Cheongju 28116, Republic of Korea; kanjon@kribb.re.kr (J.S.K.); z7v8@kribb.re.kr (J.-W.Y.); 4 Department of Marine Biotechnology, University of Science and Technology (UST), 217 Gajungro, Yuseong-gu, Daejeon 34113, Republic of Korea

**Keywords:** marine fungus, natural product, *Meira* sp., indanone, catechol, meirol, DPPH radical scavenging, α-glucosidase inhibitor, cytotoxicity

## Abstract

Three new catecholic compounds, named meirols A–C (**2**–**4**), and one known analog, argovin (**1**), were isolated from the marine-derived fungus *Meira* sp. 1210CH-42. Their structures were determined by extensive analysis of 1D, 2D NMR, and HR-ESIMS spectroscopic data. Their absolute configurations were elucidated based on ECD calculations. All the compounds exhibited strong antioxidant capabilities with EC_50_ values ranging from 6.01 to 7.47 μM (ascorbic acid, EC_50_ = 7.81 μM), as demonstrated by DPPH radical scavenging activity assays. In the α-glucosidase inhibition assay, **1** and **2** showed potent in vitro inhibitory activity with IC_50_ values of 184.50 and 199.70 μM, respectively (acarbose, IC_50_ = 301.93 μM). Although none of the isolated compounds exhibited cytotoxicity against one normal and six solid cancer cell lines, **1** exhibited moderate cytotoxicity against the NALM6 and RPMI-8402 blood cancer cell lines with GI_50_ values of 9.48 and 21.00 μM, respectively. Compound **2** also demonstrated weak cytotoxicity against the NALM6 blood cancer cell line with a GI_50_ value of 29.40 μM.

## 1. Introduction

Small-molecule natural products, such as catechol and β-lactam, are widely utilized in the drug discovery process in various ways [1,2]. Because of their low weight, small-molecule drugs can traverse cell membranes, interact with proteins and enzymes within cells, and disrupt specific processes. Alternatively, a biologically active natural product may inspire the discovery of clinically useful drug agents by offering insights into types of structural features that may prove valuable. Catechols, benzene derivatives containing a 3,4-dihydroxyphenyl group, have recently been discovered as diverse derivatives in microorganisms, plants, insects, and marine organisms [3]. Their ubiquity can be attributed to their rich redox chemistry, ability to cross-link through complex and irreversible oxidation mechanisms, excellent chelating properties, and the various ways in which vicinal hydroxyl groups interact with surfaces of significantly diverse chemical and physical characteristics [4,5]. Aligned with their structural variety, catecholamines have been reported to exhibit a broad spectrum of biological activities, encompassing antiparasitic, antibacterial, metal-chelating, anti-inflammatory, immune-modulation, wound-healing, antioxidant, neuroprotective, nephroprotective, and metabolic regulation activities [3]. Hence, novel catechol compounds derived from natural sources will serve as a promising foundation for broadening their potential biological and medical applications.

The novel basidiomycetous fungus *Meira* was first isolated from citrus leaves and mite cadavers in 2003 and assigned to *M. geulakonigii* and *M. argovae* [6]. Six species in the genus *Meira* have been reported to date, namely, *M. argovae*, *M. geulakonigii* (Boekhout et al. 2003), *M. nashicola* (Yasuda et al. 2006), *M. miltonrushii* (Rush et al. 2013), *M. siamensis* (Limtong et al. 2017), and *M. nicotianae* (Yichen et al. 2018) [6,7,8,9]. These species have been isolated from plant tissues, fruits, and mites. These *Meira* species exhibit promising antagonistic activities against a wide spectrum of fungal and bacterial plant diseases [10]. However, only one compound, argovin (**1**), has been reported from the genus *Meira* [11]. Thus, information about the active constituents responsible for the various biological activities of *Meira* sp. is still limited.

In 2023, the first marine-derived *Meira* sp. was described in our previous report as *Meira* sp. 1210CH-42 [12]. We identified new thiolactones and steroids from the *Meira* sp. strain, which exhibited α-glucosidase inhibitory activities [12]. In our ongoing efforts to find new bioactive small compounds, we have paid attention to the fungus *Meira* because the genus has not been studied well, and the strain was the first marine-derived species showing good bioactivities in our preliminary screening. Therefore, a mass culture was conducted to isolate bioactive secondary metabolites from the strain. As a result, we discovered novel bioactive catecholic compounds from the culture extract of the fungus *Meira* sp. 1210CH-42. Herein, we describe the structure determination of catecholic compounds (**1**–**4**), along with their antioxidant, α-glucosidase inhibitory, cytotoxic, tyrosinase inhibitory, and antimicrobial activities.

## 2. Results and Discussion

### 2.1. Structure Elucidation of New Compounds

**Figure 1:**
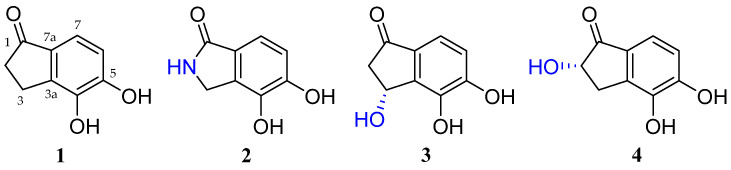
Structures of **1**–**4** from the marine fungus strain *Meira* sp. 1210CH-42.

Compound **1** was isolated as a white amorphous powder, and its molecular formula was determined to be C_9_H_8_O_3_ based on an HR-ESIMS analysis at *m*/*z* 165.0552 [M + H]^+^ (calcd for C_9_H_9_O_3_^+^, 165.0552), with six degrees of unsaturation. The ^1^H and ^13^C NMR data of **1** are summarized in Table 1 and Table 2. The ^1^H NMR spectrum of **1** in CD_3_OD revealed two olefinic protons (δ_H_ 7.15 and 6.84) and two methylene protons (δ_H_ 3.01 and 2.63). The ^13^C NMR and HSQC spectra showed the presence of nine signals, including one carbonyl carbon (δ_C_ 209.1), two oxygen-bearing sp^2^ (δ_C_ 153.1 and 143.1), two non-protonated sp^2^ (δ_C_ 144.7 and 130.8), two protonated sp^2^ (δ_C_ 117.2 and 116.9), and two methylene (δ_C_ 37.6 and 23.3) carbons. The structure of **1** was identified as argovin (4,5-dihydroxyindan-1-one) by an analysis of its NMR and HRMS data and a comparison of its spectroscopic data with those previously reported in the literature (Figure 1) [11].

**Figure 2:**
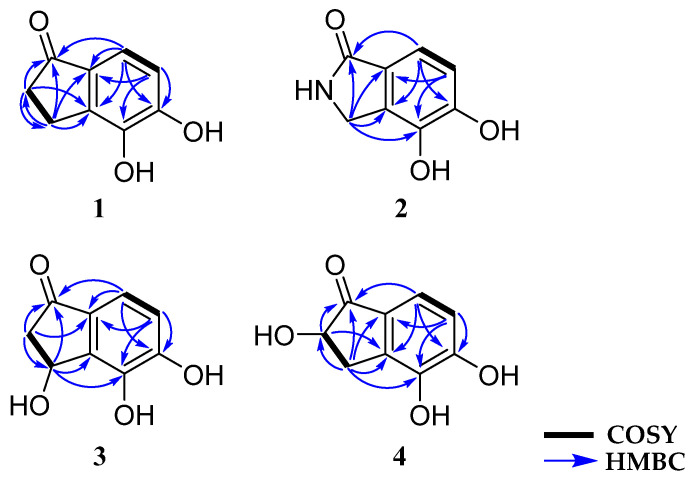
Key ^1^H-^1^H COSY and HMBC correlations of **1**–**4**.

Compound **2** was purified as a white amorphous powder. The molecular formula of **2** was determined to be C_8_H_7_NO_3_ by HR-ESIMS analysis at *m*/*z* 188.0324 [M + Na]^+^ (calcd for C_8_H_7_NO_3_Na^+^, 188.0324), which was determined to possess six degrees of unsaturation. The ^1^H NMR spectrum of **2** displayed the signals for two olefinic protons (δ_H_ 7.19 and 6.90) and two methylene protons (δ_H_ 4.34, overlapped) (Table 1). The ^13^C NMR and HSQC spectra exhibited one carbonyl (δ_C_ 174.5), two oxygen-bearing sp^2^ (δ_C_ 150.4 and 141.6), two non-protonated sp^2^ (δ_C_ 132.4 and 125.2), two protonated sp^2^ (δ_C_ 116.9 and 116.5), and one methylene (δ_C_ 44.5) carbons (Table 2). The ^1^H-^1^H COSY correlation of H-6 (δ_H_ 6.90)/H-7 (δ_H_ 7.16) revealed a pair of ortho-coupled aromatic protons at δ_H_ 6.90, d (*J* = 8.0 Hz, δ_C_ 116.9)/δ_H_ 7.16, d (*J* = 8.0 Hz, δ_C_ 116.5). Furthermore, the HMBC correlations, from H-3 (δ_H_ 4.34) to C-1 (δ_C_ 174.5)/C-3a (δ_C_ 132.4)/C-4 (δ_C_ 141.6)/C-7a (δ_C_ 125.2); from H-6 (δ_H_ 6.90) to C-4 (δ_C_ 141.6)/C-7a (δ_C_ 125.2); and from H-7 (δ_H_ 7.16) to C-1 (δ_C_ 174.5)/C-3a (δ_C_ 132.4)/C-5 (δ_C_ 150.4), indicated the presence of a 1-indanone ring system. Additionally, the HMBC correlation of the singlet at H-3 (δ_H_ 4.34, s) with a carbonyl carbon C-1 (δ_C_ 174.5) confirmed the connectivity of an amide carbonyl to the benzene ring (Figure 2). Thus, the structure of **2** was elucidated as a previously unreported catecholic compound, 4,5-dihydroxyisoindolin-1-one, and **2** was named meirol A.

**Figure 3:**
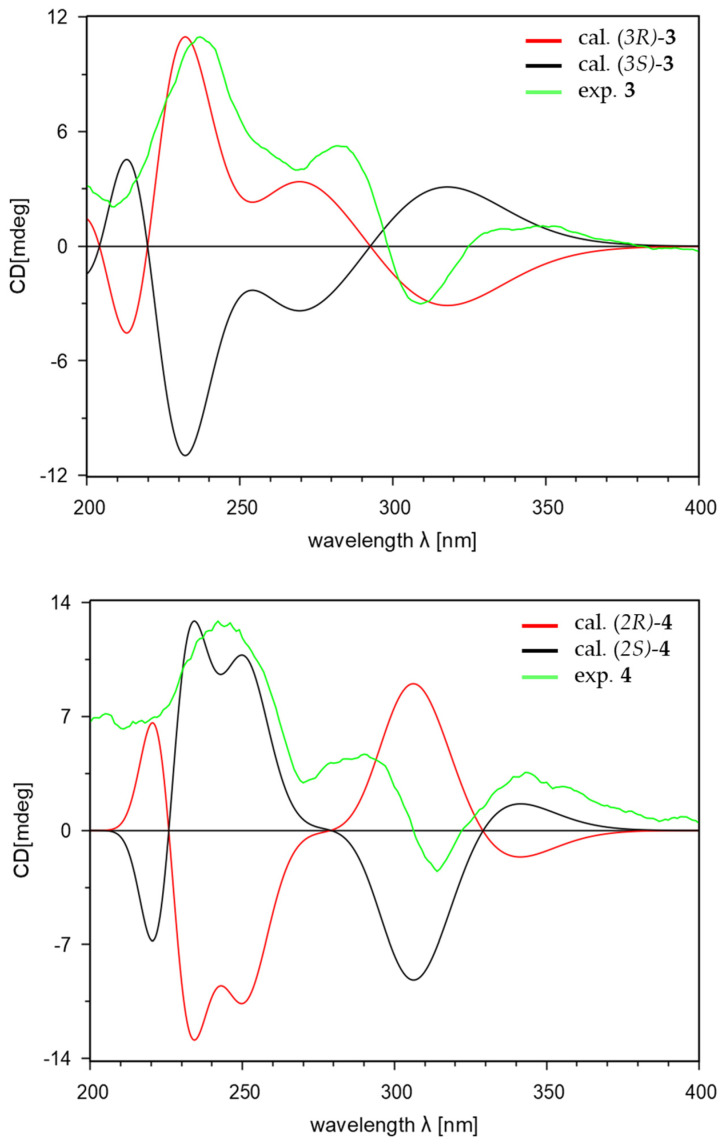
Experimental and calculated ECD spectra of **3** and **4**.

Compound **3** was obtained as a purple amorphous powder. The molecular formula of **3** was analyzed for C_9_H_8_O_4_ on the basis of its parent ion in the HR-ESIMS analysis at *m*/*z* 203.0321 [M + Na]^+^ (calcd for C_9_H_8_O_4_Na^+^, 203.0320), which required six degrees of unsaturation. The ^1^H NMR spectrum of **3** displayed signals of two olefinic protons (δ_H_ 7.14 and 6.91), one oxygenated methine proton (δ_H_ 5.48), and two methylene protons (δ_H_ 3.02 and 2.47) (Table 1). In total, nine carbon resonances were observed in the ^13^C NMR spectrum of **3**. These resonances were assigned to one carbonyl (δ_C_ 205.6), two oxygen-bearing sp^2^ (δ_C_ 154.2 and 130.0), two non-protonated sp^2^ (δ_C_ 144.1 and 143.5), two protonated sp^2^ (δ_C_ 118.6 and 116.7), one oxymethine (δ_C_ 66.9), and one methylene (δ_C_ 48.4) carbons, with the assistance of the HSQC spectrum (Table 2). Analysis of the ^1^H-^1^H COSY spectrum led to the construction of an ortho-coupled (*J* = 8.1) isolated two-proton spin system illustrating homonuclear coupling correlation between H-6 (δ_H_ 6.91, d, *J* = 8.1) and H-7 (δ_H_ 7.14, d, *J* = 8.1). The HMBC correlations from H-6 (δ_H_ 6.91) to C-4 (δ_C_ 130.0)/C-5 (δ_C_ 154.2)/C-7a (δ_C_ 144.1) and from H-7 (δ_H_ 7.14) to C-5 (δ_C_ 154.2)/C-7a (δ_C_ 144.1) confirmed the position of the spin system within an aromatic ring fragment. Additionally, the HMBC correlations from H-2 (δ_H_ 3.02 and 2.47) to C-1 (δ_C_ 205.6)/C-3 (δ_C_ 66.9)/C-7a (δ_C_ 144.1); from H-3 (δ_H_ 5.48) to C-1 (δ_C_ 205.6)/C-3a (δ_C_ 143.5)/C-4 (δ_C_ 130.0); and from H-7 (δ_H_ 7.14) to C-1 (δ_C_ 205.6) allowed for the assembly of a 1-indanone ring system. Furthermore, the presence of a hydroxy group located at C-3 (δ_C_ 66.9) was elucidated through the HMBC correlations from H-3 (δ_H_ 5.48) to C-1 (δ_C_ 205.6)/C-3a (δ_C_ 143.5)/C-4 (δ_C_ 130.0). Therefore, the planar structure of **3** was elucidated, as shown in Figure 2. To determine the absolute configuration of **3**, the theoretical electronic circular dichroism (ECD) spectra of **3** and its enantiomer were calculated (Figure 2). The experimental ECD spectrum of **3** showed good agreement with the calculated ECD spectrum of (3*R*)-**3** (Figure 3). Thus, the absolute configuration of **3** was determined as 3*R*, and the new indanone derivative, **3**, was given the name meirol B.

Compound **4** was isolated as a light purple amorphous powder. The molecular formula of **4** was determined to be identical to that of 3 (C_9_H_8_O_4_), as evidenced by the ion at *m*/*z* 203.0322 [M + Na]^+^ (calcd for C_9_H_8_O_4_Na^+^, 203.0320) from the HR-ESIMS analysis, implying six degrees of unsaturation. However, its ^1^H and ^13^C NMR spectra showed different chemical shifts compared with **3**. The ^1^H NMR spectrum of **4** (Table 1) showed the signals for two olefinic protons (δ_H_ 7.17 and 6.87), one oxygenated methine proton (δ_H_ 4.41), and two methylene protons (δ_H_ 3.51 and 2.70). The ^13^C NMR and HSQC spectra of **4** exhibited the presence of nine carbon resonances, including one carbonyl (δ_C_ 207.0), two oxygen-bearing sp^2^ (δ_C_ 153.8 and 128.1), two non-protonated sp^2^ (δ_C_ 143.0 and 139.8), two protonated sp^2^ (δ_C_ 118.0 and 117.3), one oxymethine (δ_C_ 75.0), and one methylene (δ_C_ 32.2) carbons (Table 2). The ^1^H-^1^H COSY correlation of H-2 (δ_H_ 4.41)/H-3 (δ_H_ 3.51 and 2.70) and H-6 (δ_H_ 6.87)/H-7 (δ_H_ 7.17) and the strong HMBC correlations from H-2 (δ_H_ 4.41) to C-1 (δ_C_ 207.0)/C-3a (δ_C_ 139.8) and from H-3 (δ_H_ 3.51 and 2.70) to C-1 (δ_C_ 207.0)/C-2 (δ_C_ 75.0)/C-3a (δ_C_ 139.8)/C-4 (δ_C_ 128.1)/C-7a (δ_C_ 143.0) indicated that a hydroxy group was located at C-2 (δ_C_ 75.0), and two oxygenated quaternary carbons were located at C-4 (δ_C_ 128.1) and C-5 (δ_C_ 153.8). In addition, the presence of a 1-indanone system was supported by the ^1^H-^1^H COSY correlation between H-6 (δ_H_ 6.87, d, *J* = 8.1) and H-7 (δ_H_ 7.17, d, *J* = 8.1), as well as the HMBC correlations from H-6 (δ_H_ 6.87) to C-4 (δ_C_ 128.1)/C-5 (δ_C_ 153.8)/C-7a (δ_C_ 143.0) and from H-7 (δ_H_ 7.17) to C-1 (δ_C_ 207.0)/C-3a (δ_C_ 139.8)/C-5 (δ_C_ 153.8). Based on these data analyses, the planar structure of **4** was determined to be a new positional isomer of **3** (Figure 2). The absolute configuration of **4** was defined by comparing its calculated and experimental ECD spectra. As shown in Figure 3, the calculated ECD spectrum of 2*S*-**4** closely matched the experimental spectrum, confirming the 2*S* absolute configuration for **4**. Therefore, the structure of the new natural product, **4**, was clearly determined, and **4** was named meirol C.

### 2.2. Bioactivity Evaluation of Compounds

The antioxidant activities of **1**–**4** were assessed using the 1,1-diphenyl-2-picrylhydrazyl (DPPH) radical scavenging assay. As indicated in Table 3, **1**–**4** exhibited considerable free radical scavenging activities with EC_50_ values ranging from 6.01 ± 0.07 to 7.47 ± 0.13 μM, showing better activities than the positive control, ascorbic acid (EC_50_ = 7.81 ± 0.25 μM). Also, **1**–**4** were evaluated for α-glucosidase inhibitory activities (Table 3). Compounds **1** and **2** exhibited significant inhibitory effects with IC_50_ values of 184.50 ± 2.93 and 199.70 ± 1.87 μM, respectively. Meanwhile, **3** showed weaker activity (IC_50_ = 367.43 ± 3.01 μM) than the positive control, acarbose (IC_50_ = 301.93 ± 3.55 μM). A structure–activity relationship analysis of **1**–**4** indicated that the hydroxy groups at C-2 or C-3 in diol-indanone impacted their antioxidant properties but did not have a significant impact on their α-glucosidase inhibitory activity. Furthermore, all the compounds were screened for their cytotoxic activity against six solid and seven blood cancer cell lines (Table 3). Compounds **1** and **2** showed selective cytotoxicity against two out of the seven blood cancer cell lines (HL-60, acute myelogenous leukemia; Raji, Burkitt’s lymphoma; K562, chronic myelogenous leukemia; RPMI-8402, T cell acute lymphocytic leukemia; NALM6, B cell acute lymphocytic leukemia; U266, multiple myeloma; WSU-DLCL2, diffuse large B cell lymphoma). Among the tested compounds, only **1** showed weak cytotoxicity (GI_50_ = 21.00 ± 0.47 μM) against the RPMI-8402 cell line. Compounds **1** and **2** exhibited selective cytotoxicity against the NALM6 blood cancer cell line (GI_50_ = 9.47 ± 0.41 and 29.55 ± 2.27 μM, respectively), while the other compounds did not demonstrate significant cytotoxicity (GI_50_ > 30 μM). Additionally, all compounds showed no cytotoxicity against one normal cell (MRC-9) and six solid cancer cell lines (PC-3, prostate; NCI-H23, lung; HCT-15, colon; NUGC-3, stomach; ACHN, renal; MDA-MB-231, breast) even at a concentration of 30 μM.

Compounds **1**–**4** did not show significant tyrosinase inhibitory activity at a concentration of 100 μM (kojic acid, IC_50_ = 41.85 μM). Also, **1**–**4** were evaluated for their antimicrobial properties against three Gram-positive bacteria (*Bacillus subtilis* KCTC 1021, *Micrococcus luteus* KCTC 1915, and *Staphylococcus aureus* KCTC 1927) and three Gram-negative bacteria (*Escherichia coli* KCTC 2441, *Salmonella typhimurium* KCTC 2515, and *Klebsiella pneumonia* KCTC 2690). However, none of the compounds inhibited the growth of Gram-positive and Gram-negative bacteria at a concentration of 32.0 μg/mL.

## 3. Materials and Methods

### 3.1. General Experimental Procedures and Reagents

NMR spectra were acquired with a Bruker AVANCE III 600 spectrometer (Bruker Biospin GmbH, Rheinstetten, Germany) with a 3 mm probe operating at 600 MHz (^1^H) and 150 MHz (^13^C). Chemical shifts were expressed in ppm with reference to the solvent peaks (δ_H_ 3.31 and δ_C_ 49.15 ppm for CD_3_OD). UV spectra were recorded with a Shimadzu UV-1650PC spectrophotometer (Shimadzu Corporation, Kyoto, Japan). IR spectra were obtained on an OPUS FT/IR-ALPHA II spectrophotometer (Bruker OPTIK GmbH & Co. KG, Ettlingen, Germany). Optical rotations were measured with a Rudolph analytical Autopol III S2 polarimeter (Rudolph Research Analytical, Hackettstown, NJ, USA). LR-ESIMS data were obtained with an ISQ EM mass spectrometer. HR-ESIMS data were obtained with a Waters SYNPT G2 Q-TOF mass spectrometer (Waters Corporation, Milford, MA, USA) at the Korea Basic Science Institute (KBSI) in Cheongju, Republic of Korea. ECD spectra were recorded with a JASCO J-1500 circular dichroism spectrometer (JASCO Corporation, Tokyo, Japan) at the Center for Research Facilities, Changwon National University, in Changwon, Republic of Korea. HPLC was performed using a BLS-Class pump (Teledyne SSI, Inc., State College, PA 16803, USA) with a Shodex RI-201H refractive index detector (Shoko Scientific Co., Ltd., Yokohama, Japan). Columns for HPLC were YMC-Triart C_18_ (250 mm × 10 mm, 5 μm), YMC-Triart C_8_ (250 mm × 10 mm, 5 μm), and YMC-CHIRAL PREP CD PM (250 mm × 4.6 mm, S-10 μm). RP silica gel (YMC-Gel ODS-A, 12 nm, S-75 μm) was used for open-column chromatography. Organic solvents were purchased as HPLC grade, and ultrapure waters were obtained from the Milipore Mili-Q Direct 8 system. The reagents used in the bioassay were purchased from Sigma-Aldrich and Tokyo Chemical Industry. Cancer cell lines were purchased from the Japanese Cancer Research Resources Bank (JCRB) (NUGC-3, JCRB Cell Bank/Cat. # JCRB0822), the DSMZ-German Collection of Microorganisms and Cell Cultures (RPMI-8402, DSMZ/Cat # ACC 290; WSU-DLCL2, DSMZ/Cat # ACC 575), and the American Type Culture Collection (ATCC) (PC-3, ATCC/Cat. # CRL-1435; MDA-MB-231, ATCC/Cat. # HTB-26; ACHN, ATCC/Cat. # CRL-1611; NCI-H23, ATCC/Cat. # CRL-5800; HCT-15, ATCC/Cat. # CCL-225; HL-60, ATCC/Cat. # CCL-240; Raji, ATCC/Cat # CCL-86; K562, ATCC/Cat # CCL-243; NALM6, ATCC/Cat # CRL-3273; U266, ATCC/Cat # TIB-196).

### 3.2. Fungal Strain and Fermentation

The fungal strain *Meira* sp. 1210CH-42 was obtained from a seawater sample collected at the Chuuk Islands, Federated States of Micronesia, in 2010, as described previously [12]. The strain was identified as *Meira* sp. (GenBank accession number OQ693946) through DNA amplification and by sequencing the ITS region of the rRNA gene, as described earlier [12]. The cultures of strain 1210CH-42 were performed in modified Bennett’s broth medium (1% D-glucose, 0.2% tryptone, 0.1% yeast extract, 0.1% beef extract, 0.5% glycerol, sea salt 10 g/L, pH 7.0). A seed culture was prepared from a spore suspension of strain 1210CH-42 by inoculating it into 2 L flasks and incubating it at 28 °C for 7 days in a rotary shaker at 120 rpm. The seed culture was inoculated aseptically into a 100 L fermenter containing 70 L of sterilized culture medium (0.1% *v*/*v*). Large-scale fermentation was conducted at 28 °C, 40 rpm, and with an airflow rate of 10 L/min (LPM) for a duration of 21 days before being harvested.

### 3.3. Extraction and Isolation of Compounds ***1***–***4***

A culture broth of strain 1210CH-42 (total, 70 L) was harvested via high-speed centrifugation at 60,000 rpm. Subsequently, the supernatant was extracted two times with ethyl acetate (EtOAc, 140 L). The EtOAc layer was evaporated under reduced pressure to yield a crude extract (4.61 g). The crude extract was subjected to ODS open column chromatography (YMC Gel ODS-A, 12 nm, S75 μm) followed by stepwise gradient elution with MeOH/H_2_O (*v*/*v*) (20:80, 40:60, 60:40, 80:20, and 100:0) as an eluent. The 20% MeOH (0.89 g) was subjected to ODS open column chromatography (YMC Gel ODS-A, 12 nm, S75 μm) followed by stepwise gradient elution with MeOH/H_2_O (*v*/*v*) (5:95, 10:90, 15:85, 20:80, and 100:0) to provide five fractions (F1–F5). Fraction F4 (293 mg) was purified by a semi-preparative reversed-phase HPLC (YMC-Triart C_18_ column 250 mm × 10 mm i.d., 5 μm; 15% MeOH in H_2_O; flow rate: 2.0 mL/min; detector: RI) to yield **1** (18.1 mg, *t*_R_ 62.0 min). Fraction F2 (140 mg) was further purified by a semi-preparative RP HPLC (YMC-Triart C_18_ column 250 mm × 10 mm i.d., 5 μm; 5% MeOH in H_2_O; flow rate: 2.0 mL/min; detector: RI) to obtain eighteen subfractions (F2.SF1–F2.SF18). Compound **2** was re-purified from subfraction F2.SF16 by a semi-preparative RP HPLC (YMC-Triart C_8_ column 250 mm × 10 mm i.d., 5 μm; 5% MeOH in H_2_O; flow rate: 2.0 mL/min; detector: RI) to yield **2** (8.2 mg, *t*_R_ 38.0 min). Fraction F3 (266 mg) was purified using a semi-preparative RP HPLC (YMC-Triart C_18_ column 250 mm × 10 mm i.d., 5 μm; 10% MeOH in H_2_O; flow rate: 2.0 mL/min; detector: RI) to yield eighteen subfractions (F3.SF1–F3.SF18). Subsequently, compounds **3** and **4** were isolated from subfraction F3.SF4 using an analytical HPLC (YMC-CHIRAL PREP CD PM 250 mm × 4.6 mm, S-10 μM; flow rate: 0.8 mL/min; detector: RI) with an isocratic elution of 5% MeOH in H_2_O to yield **3** (4.3 mg, *t*_R_ 11.0 min) and **4** (5.2 mg, *t*_R_ 13.0 min).

Argovin (**1**): White amorphous powder; UV (MeOH) *λ*_max_ (log *ε*) 213 (3.71), 236 (3.95), 283 (3.82) nm; IR (MeOH) *ν*_max_ 3200, 1651, 1589, 1471, 1299 cm^−1^; ^1^H and ^13^C NMR data (CD_3_OD), see Table 1 and Table 2; HR-ESIMS *m*/*z* 165.0552 [M + H]^+^ (calcd. for C_9_H_9_O_3_^+^, 165.0552; 187.0371) and [M + Na]^+^ (calcd for C_9_H_8_O_3_Na^+^, 187.0371).

Meirol A (**2**): White amorphous powder; UV (MeOH) *λ*_max_ (log *ε*) 219 (3.96), 261 (3.83), 290 (3.39) nm; IR (MeOH) *ν*_max_ 3233, 1626, 1448, 1419, 1292 cm^−1^; ^1^H and ^13^C NMR data (CD_3_OD), see Table 1 and Table 2; HR-ESIMS *m*/*z* 166.0503 [M + H]^+^ (calcd. for C_8_H_8_NO_3_^+^, 166.0504; 188.0324) and [M + Na]^+^ (calcd for C_8_H_7_NO_3_Na^+^, 188.0324).

Meirol B (**3**): Purple amorphous powder; ${\left[\alpha \right]}_{D}^{25}$ +23.3 (*c* 0.1, MeOH); UV (MeOH) *λ*_max_ (log *ε*) 214 (3.81), 235 (3.98), 287 (3.74) nm; ECD (MeOH, *λ* [nm] (Δ*ε*), *c* = 1.58 mM) 309 (−0.83), 282 (+1.44), 271 (+1.10), 237 (+3.00), 209 (0.56); IR (MeOH) *ν*_max_ 3270, 1681, 1592, 1488, 1280 cm^−1^; ^1^H and ^13^C NMR data (CD_3_OD), see Table 1 and Table 2; HR-ESIMS *m*/*z* 203.0321 [M + Na]^+^ (calcd for C_9_H_8_O_4_Na^+^, 203.0320).

Meirol C (**4**): Light purple amorphous powder; ${\left[\alpha \right]}_{D}^{25}$ +23.3 (*c* 0.1, MeOH); UV (MeOH) *λ*_max_ (log *ε*) 212 (3.72), 237 (3.95), 291 (3.77) nm; ECD (MeOH, *λ* [nm] (Δ*ε*), *c* = 1.58 mM) 343 (+0.71), 314 (−0.50), 290 (+0.94), 270 (+0.59), 242 (+2.57); IR (MeOH) *ν*_max_ 3256, 1686, 1611, 1286 cm^−1^; ^1^H and ^13^C NMR data (CD_3_OD), see Table 1 and Table 2; HR-ESIMS *m*/*z* 203.0322 [M + Na]^+^ (calcd for C_9_H_8_O_4_Na^+^, 203.0320).

### 3.4. Computational Analysis

The initial geometry optimization and conformational searches were generated using Conflex 8 (rev. B, Conflex Corp., Tokyo, Japan). The optimization and calculation for electronic circular dichroism (ECD) were conducted utilizing the Gaussian 16 program (rev. B.01, Gaussian Inc., Wallingford, CT, USA). Conformational searches were executed through MMFF94s force field calculations, with a search limit set at 5 kcal/mol. The conformers were optimized using the ground state method at the CAM-B3LYP/6-31 G+ (d, p) level in MeOH with an IEFPCM model for ECD. The theoretical calculations of ECD spectra were performed using TD-SCF at the CAM-B3LYP /6-31 G+ (d, p). The ECD spectrum was derived by calculating the Boltzmann-weighted sum of conformer spectra. The final ECD spectra were simulated using SpecDis (v. 1.71) with *σ* values ranging from 0.20 to 0.30 eV. All calculated curves were UV-shifted by +10 to +15 nm to better simulate experimental spectra.

### 3.5. Antioxidant Activity Assay

The DPPH radical scavenging activities of **1**–**4** were determined by the reported method [13]. In a 96-well plate, 100 μL of sample solution (in MeOH) was mixed with 100 μL of DPPH solution (0.16 mM in MeOH), shaken several times, and then incubated at room temperature for 30 min. The absorbance at 517 nm was recorded. Ascorbic acid was used as the positive control, and the experiments were performed in triplicate.

### 3.6. α-Glucosidase Inhibitory Activity Assay

The α-glucosidase inhibitory activity was determined by measuring the absorbance increase resulting from the hydrolysis of *p*-nitrophenyl-α-D-glucopyranoside (*p*NPG, TCI) by α-glucosidase at 405 nm using a microplate reader, according to the reference to previously reported literature [12]. The 130 μL sample solution (in 0.1 mM PBS) with the 30 μL α-glucosidase solution (0.2 U/mL) was incubated at 37 °C for 10 min. Subsequently, 40 μL of 5 mM *p*NPG was added. The reaction mixture was further incubated at 37 °C for 20 min. The α-glucosidase inhibitory activity was determined using a microplate reader at 405 nm. The negative control was prepared by substituting PBS buffer for the sample in the same way as the test. Acarbose served as the positive control, and the experiments were conducted in triplicate.

### 3.7. Cytotoxicity Assay

The cytotoxic activities of **1**–**4** were measured using the CellTiter-Glo luminescent cell viability assay (Promega, Madison, WI, USA) and conducted by the SRB (sulforhodamine B) assay, as previously described [14,15]. The luminescence signal was quantified using a GloMax-Multi Detection System (Promega, Madison, WI, USA), and GI_50_ values were determined utilizing a relative GI_50_ model in GraphPad Prism (GraphPad, San Diego, CA, USA). Doxorubicin was used as the positive control.

### 3.8. Tyrosinase Inhibitory Activity Assay

The tyrosinase inhibitory activity was assessed using L-DOPA and the 96-well microplate method, as previously reported [16]. Briefly, 140 μL of 20 mM phosphate buffer (pH 6.8) and 20 μL of mushroom tyrosinase (480 U/mL) in the same buffer were added to wells containing 20 μL of the test compounds. After incubation for 10 min at 25 °C, 20 μL of L-DOPA (0.85 mM) was added to the 200 μL reaction system. The incubation was continued for another 20 min at 25 °C; then, the colored end product’s absorbance was measured at 475 nm using a microplate reader. Kojic acid was utilized as a positive control in the reference sample experiment. All experiments were performed in triplicate for each compound.

### 3.9. Antibacterial Assay

The antibacterial activities were determined using the 96-well microplate method, as described in a published report, against three Gram-positive and three Gram-negative bacteria [17].

## 4. Conclusions

In summary, four catecholic compounds (**1**−**4**), including three new ones (**2**–**4**), were identified from *Meira* sp. 1210CH-42. Their structures were elucidated by a detailed analysis of NMR and HRESIMS data. The absolute configurations of **3** and **4** were determined by calculating the ECD spectra of their possible isomers. The antioxidant-DPPH assay showed that all compounds exhibited more significant free radical scavenging activity (EC_50_ = 6.01–7.47 μM) than ascorbic acid. Additionally, **1** and **2** exhibited moderate α-glucosidase inhibitory activity (IC_50_ = 184.50 and 199.70 μM, respectively) and selective cytotoxicity against blood cancer cell lines (RPMI-8402 and NALM6, GI_50_ = 9.47–29.40 μM). As a result, the catecholic indanones from *Meira* sp. 1210CH-42 could serve as a potential agent for antioxidative, α-glucosidase inhibitory, and anticancer leads. Further investigation is needed to determine the biological mechanism of compounds from the marine-derived fungus *Meira*.

## Figures and Tables

**Table 1:** ^1^H NMR data of **1**–**4** (600 MHz for ^1^H in CD_3_OD).

Position	1	2	3	4
	δ_H_, Mult (*J* in Hz)	δ_H_, Mult (*J* in Hz)	δ_H_, Mult (*J* in Hz)	δ_H_, Mult (*J* in Hz)
2	2.63, t (5.6)		2.47, d (18.6) 3.02, dd (18.6, 6.6)	4.41, dd (7.7, 4.5)
3	3.01, t (5.6)	4.34, s	5.48, d (6.6)	2.70, dd (16.7, 4.5) 3.51, dd (16.7, 7.7)
6	6.84, d (8.0)	6.90, d (8.0)	6.91, d (8.0)	6.87, d (8.1)
7	7.15, d (8.0)	7.16, d (8.0)	7.14, d (8.0)	7.17, d (8.1)

**Table 2:** ^13^C NMR data of **1**–**4** (150 MHz for ^13^C, in CD_3_OD).

Position	1	2	3	4
	δ_C_, Type	δ_C_, Type	δ_C_, Type	δ_C_, Type
1	209.1, C	174.5, C	205.6, C	207.0, C
2	37.6, CH_2_	NH	48.4, CH_2_	75.0, CH
3	23.3, CH_2_	44.5, CH_2_	66.9, CH	32.2, CH_2_
3a	144.7, C	132.4, C	143.5, C	139.8, C
4	143.1, C	141.6, C	130.0, C	128.1, C
5	153.1, C	150.4 C	154.2, C	153.8, C
6	116.9, CH	116.9, CH	118.6, CH	117.3, CH
7	117.2, CH	116.5, CH	116.7, CH	118.0, CH
7a	130.8, C	125.2, C	144.1, C	143.0, C

**Table 3:** Antioxidant, α-glucosidase inhibitory, and cytotoxic activities of **1**–**4**.

Compound	DPPH Radical Scavenging Activity (EC_50_ ± SD, μM) *^a^*	α-Glucosidase Inhibitory Activity (IC_50_ ± SD, μM) *^b^*	Cytotoxic Activity (GI_50_ ± SD, μM) *^c^*	
			RPMI-8402 *^d^*	NALM6 *^d^*
1	7.47 ± 0.13	184.50 ± 2.93	21.00 ± 0.47	9.47 ± 0.41
2	6.82 ± 0.06	199.70 ± 1.87	>30	29.55 ± 2.27
3	6.01 ± 0.07	367.43 ± 3.01	>30	>30
4	6.20 ± 0.12	>555.00	>30	>30
Ascorbic acid	7.81 ± 0.25	–	–	–
Acarbose	–	301.93 ± 3.55	–	–
Doxorubicin	–	–	0.015 ± 0.13 × 10^−2^	0.003 ± 0.06 × 10^−2^

*^a^* The half maximal effective concentration (μM); *^b^* the 50% inhibitory concentration (μM); *^c^* the 50% growth inhibitory concentration (μM) values represent the means ± standard deviation based on triplicate experiments; *^d^* RPMI-8402, T cell acute lymphocytic leukemia; NALM6, B cell acute lymphocytic leukemia.

## Data Availability

The data presented in the article are available in the Supplementary Materials.

## Supplementary Materials

The following are available online at https://www.mdpi.com/article/10.3390/md22020087/s1: Figures S1–S6: ^1^H, ^13^C NMR, HSQC, COSY, HMBC, and HR-ESIMS data of **1**. Figures S7–S30: ^1^H, ^13^C NMR, HSQC, COSY, HMBC, HR-ESIMS, UV, and IR data of **2**–**4**. Figures S31–S32 and Tables S1–S4: TDSCF-ECD calculation data of **3**–**4**.
