# Supplementary material for: Meirols A–C: Bioactive Catecholic Compounds from the Marine-Derived Fungus Meira sp. 1210CH-42
Source: Mar Drugs. 2024 Feb 14;22(2):87. doi: 10.3390/md22020087 (PMC10890530; doi:10.3390/md22020087)
Supplement: Supplementary file 1 [file marinedrugs-22-00087-s001.zip › marinedrugs-2861663-supplementary-1.pdf]

Supporting Information

Meirols A–C: Bioactive Catecholic Compounds from the Marine-Derived Fungus *Meira* sp. 1210CH-42

Min Ah Lee [1,2], Jong Soon Kang [3], Jeong-Wook Yang [3], Hwa-Sun Lee [1,2], Chang-Su Heo [1,4], Sun Joo Park [2], and Hee Jae Shin [1,4,*]

[1]Marine Natural Products Chemistry Laboratory, Korea Institute of Ocean Science and Technology, 385 Haeyang-ro, Yeongdo-gu, Busan 49111, Republic of Korea; minah@kiost.ac.kr (M.A.L.); hwasunlee@kiost.ac.kr (H.-S.L.)

[2]Department of Chemistry, Pukyong National University, 45 Yongso-ro, Nam-Gu, Busan 48513, Republic of Korea; parksj@pknu.ac.kr (S.J.P.)

[3]Laboratory Animal Resource Center, Korea Research Institute of Bioscience and Biotechnology, 30 Yeongudanji-ro, Cheongwon-gu, Cheongju 28116, Republic of Korea; kanjon@kribb.re.kr (J.S.K.); z7v8@kribb.re.kr (J.-W.Y.)

[4]Department of Marine Biotechnology, University of Science and Technology (UST), 217 Gajungro, Yuseong-gu, Daejeon 34113, Republic of Korea; science30@kiost.ac.kr (C.-S.H.)

*Correspondence: shinhj@kiost.ac.kr; Tel.: +82-51-664-3341; Fax: +82-51-664-3340

Contents

Figure S1. ^{1}H **NMR spectrum of 1 in CD_3OD (600 MHz).** 3
Figure S2. ^{13}C **NMR spectrum of 1 in CD_3OD (150 MHz).** 3
Figure S3. HSQC spectrum of 1 in CD_3OD. 4
Figure S4. COSY spectrum of 1 in CD_3OD. 4
Figure S5. HMBC spectrum of 1 in CD_3OD. 5
Figure S6. HR-ESIMS spectrum of 1. 6
Figure S7. ^{1}H **NMR spectrum of 2 in CD_3OD (600 MHz).** 7
Figure S8. ^{13}C **NMR spectrum of 2 in CD_3OD (150 MHz).** 7
Figure S9. HSQC spectrum of 2 in CD_3OD. 8
Figure S10. COSY spectrum of 2 in CD_3OD. 8
Figure S11. HMBC spectrum of 2 in CD_3OD. 9
Figure S12. HR-ESIMS spectrum of 2. 10
Figure S13. UV spectrum of 2. 11
Figure S14. IR spectrum of 2. 12
Figure S15. ^{1}H **NMR spectrum of 3 in CD_3OD (600 MHz).** 13
Figure S16. ^{13}C **NMR spectrum of 3 in CD_3OD (150 MHz).** 13
Figure S17. HSQC spectrum of 3 in CD_3OD. 14
Figure S18. COSY spectrum of 3 in CD_3OD. 14
Figure S19. HMBC spectrum of 3 in CD_3OD. 15
Figure S20. HR-ESIMS spectrum of 3. 16
Figure S21. UV spectrum of 3. 17
Figure S22. IR spectrum of 3. 18
Figure S23. ^{1}H **NMR spectrum of 4 in CD_3OD (600 MHz).** 19
Figure S24. ^{13}C **NMR spectrum of 4 in CD_3OD (150 MHz).** 19
Figure S25. HSQC spectrum of 4 in CD_3OD. 20
Figure S26. COSY spectrum of 4 in CD_3OD. 20
Figure S27. HMBC spectrum of 4 in CD_3OD. 21
Figure S28. HR-ESIMS spectrum of 4. 22
Figure S29. UV spectrum of 4. 23
Figure S30. IR spectrum of 4. 24
Figure S31. Initial geometry optimized conformers of 3. 25
Figure S32. Initial geometry optimized conformers of 4. 26

Table S1. Total Gibbs Free Energy and Boltzmann population of initial geometry optimized conformers of 3. 25
Table S2. The cartesian coordinates of conformers of 3. 25
Table S3. Total Gibbs Free Energy and Boltzmann population of initial geometry optimized conformers of 4. 26
Table S4. The cartesian coordinates of conformers of 4. 27

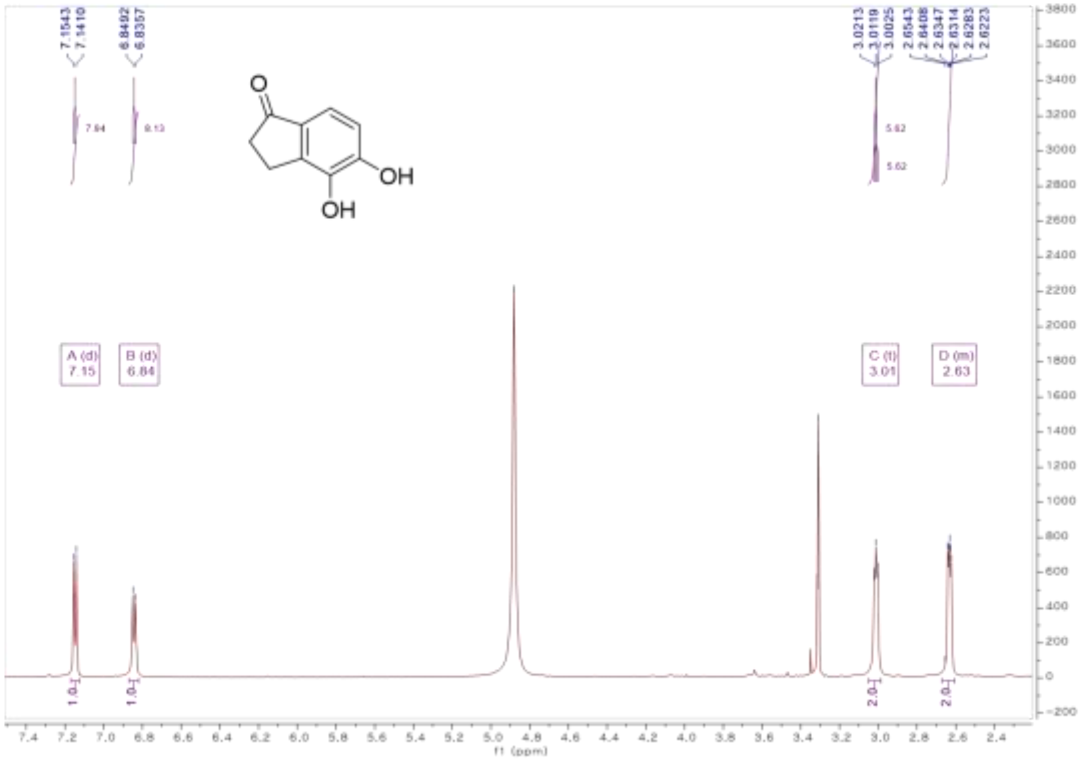

Figure S1. ^{1}H NMR spectrum of **1** in CD_3OD (600 MHz).

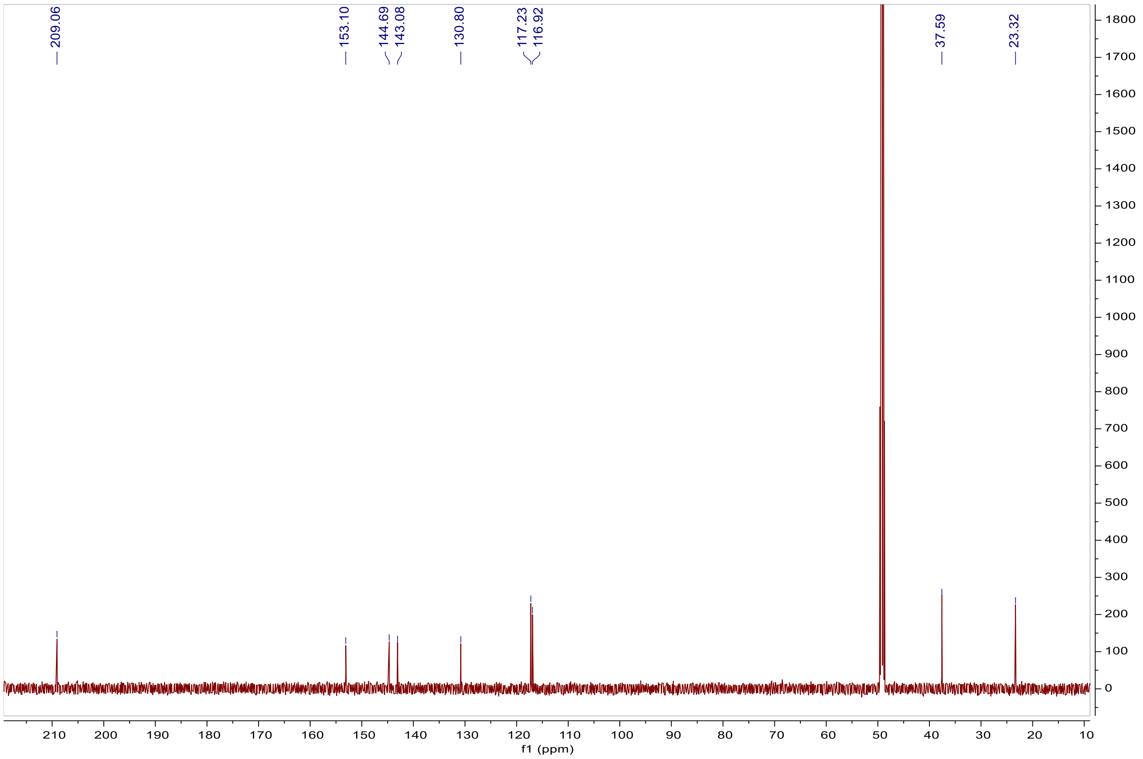

Figure S2. ^{13}C NMR spectrum of **1** in CD_3OD (150 MHz).

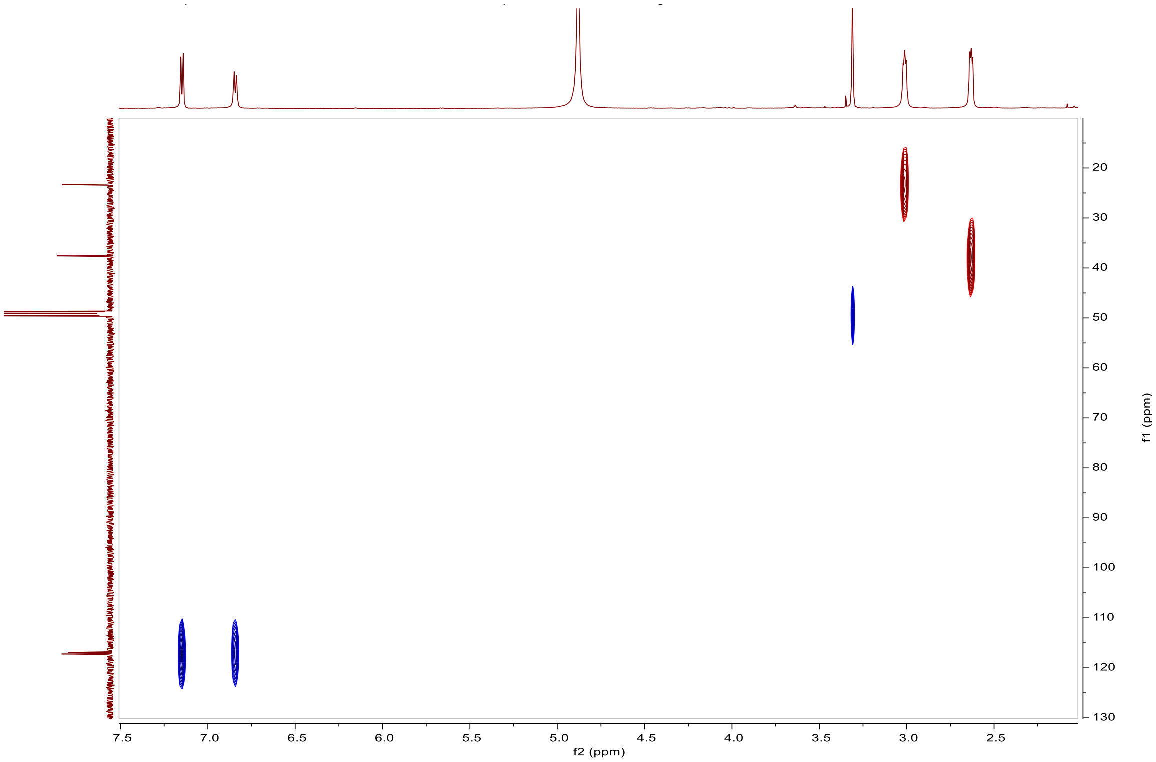

Figure S3. HSQC spectrum of **1** in CD_3OD.

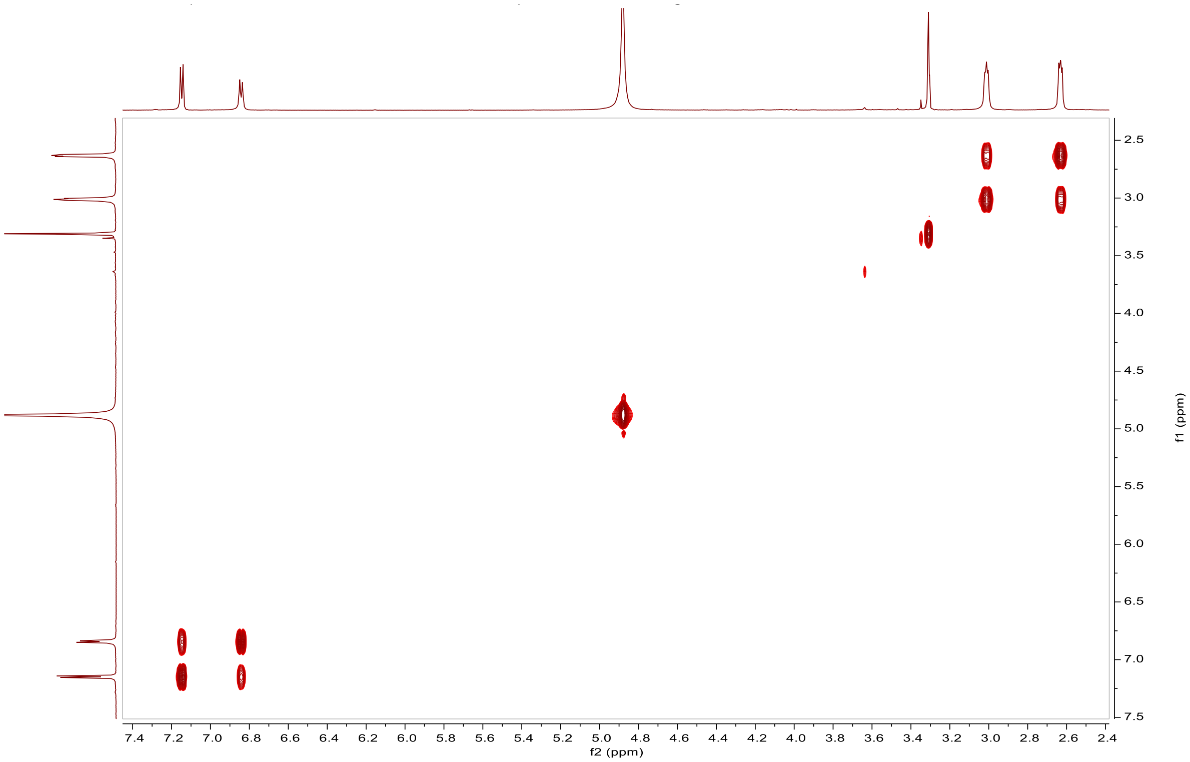

Figure S4. COSY spectrum of **1** in CD_3OD.

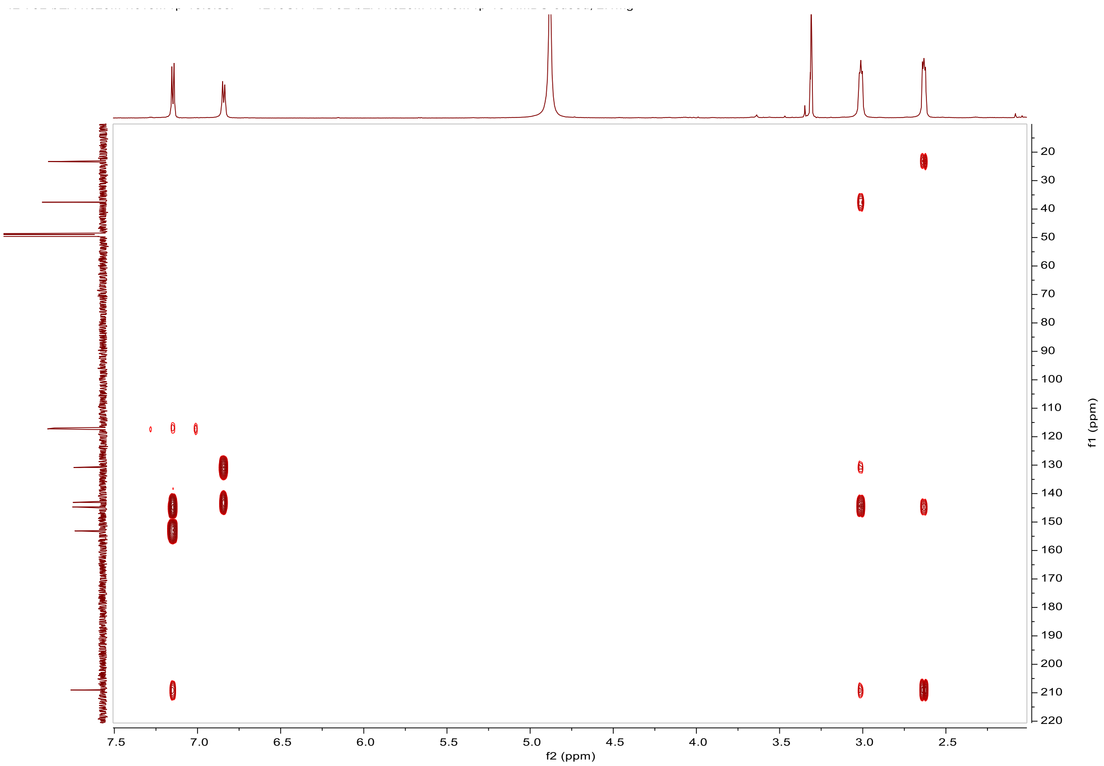

Figure S5. HMBC spectrum of **1** in CD_3OD.

Sample : 01. 2308-M-A1
(+) ESI-MS
Range : 100-250 m/z

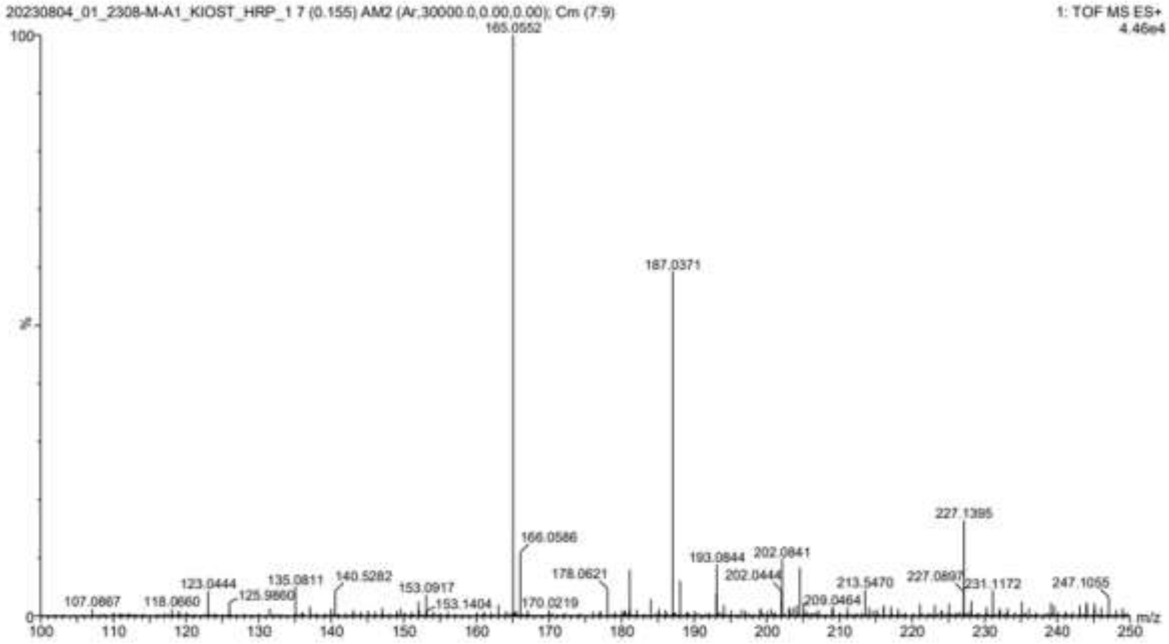

Elemental Composition Report

Single Mass Analysis
Tolerance = 10.0 PPM / DBE: min = -1.5, max = 50.0
Element prediction: Off
Number of isotope peaks used for i-FIT = 3

Monoisotopic Mass, Even Electron Ions
Elements Used:
C: 0-10 H: 0-300 O: 0-5 Na: 0-1 Pt: 0-1

Minimum:				-1.5				
Maximum:		5.0	10.0	50.0				
Mass	Calc. Mass	mDa	PPM	DBE	i-FIT	Norm	Conf(%)	Formula
165.0552	165.0552	0.0	0.0	5.5	990.7	n/a	n/a	C9 H9 O3
187.0371	187.0371	0.0	0.0	5.5	811.3	n/a	n/a	C9 H8 O3 Na

Figure S6. HR-ESIMS spectrum of **1**.

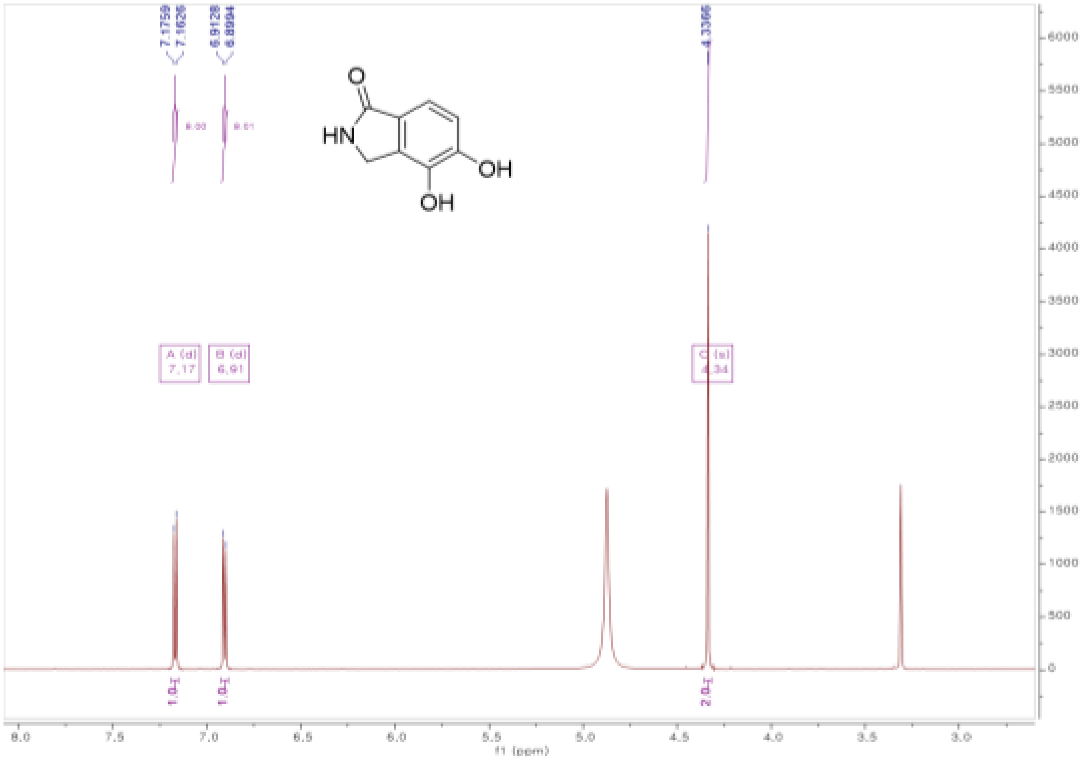

Figure S7. ^{1}H NMR spectrum of **2** in CD_3OD (600 MHz).

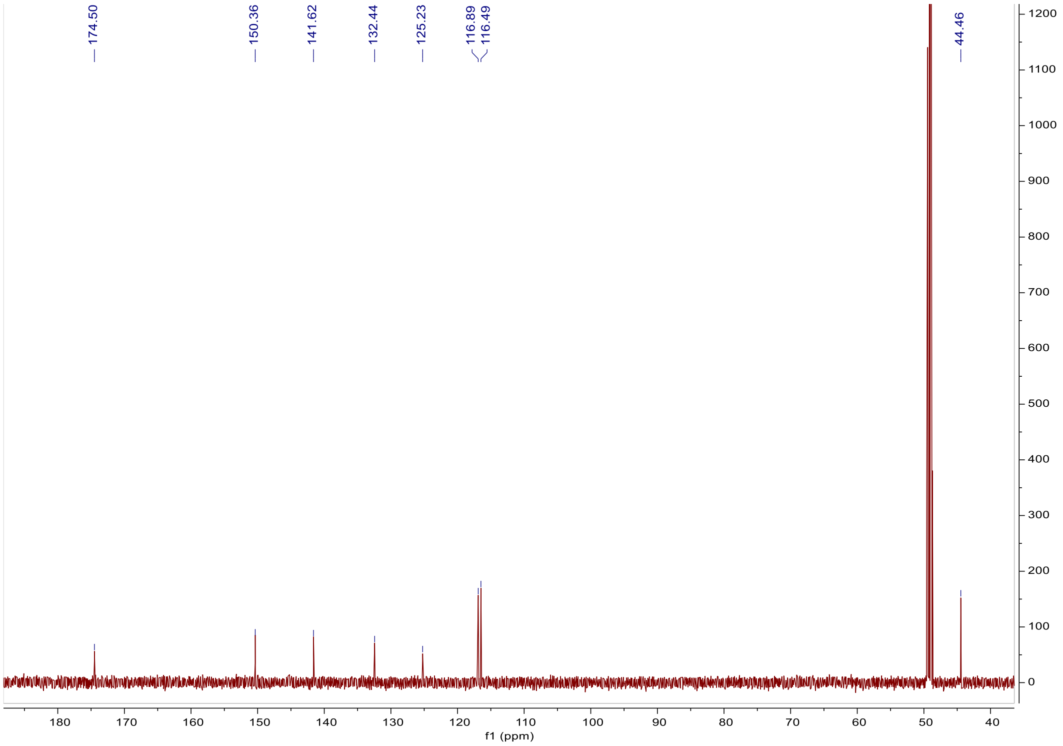

Figure S8. ^{13}C NMR spectrum of **2** in CD_3OD (150 MHz).

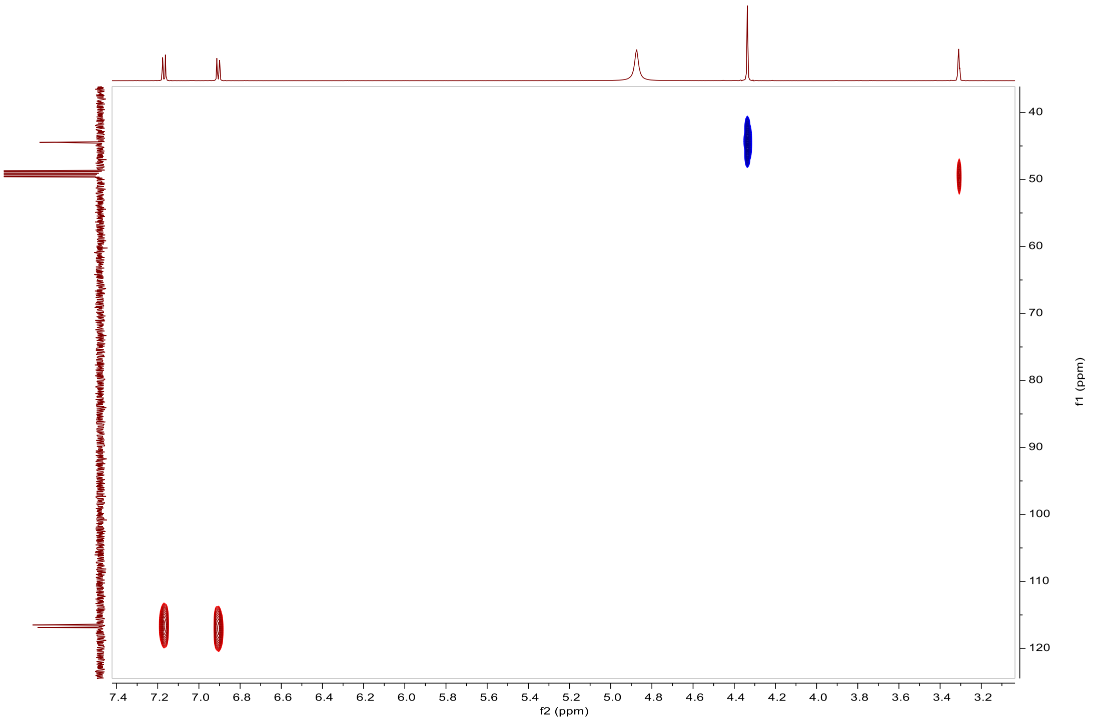

Figure S9. HSQC spectrum of **2** in CD_3OD.

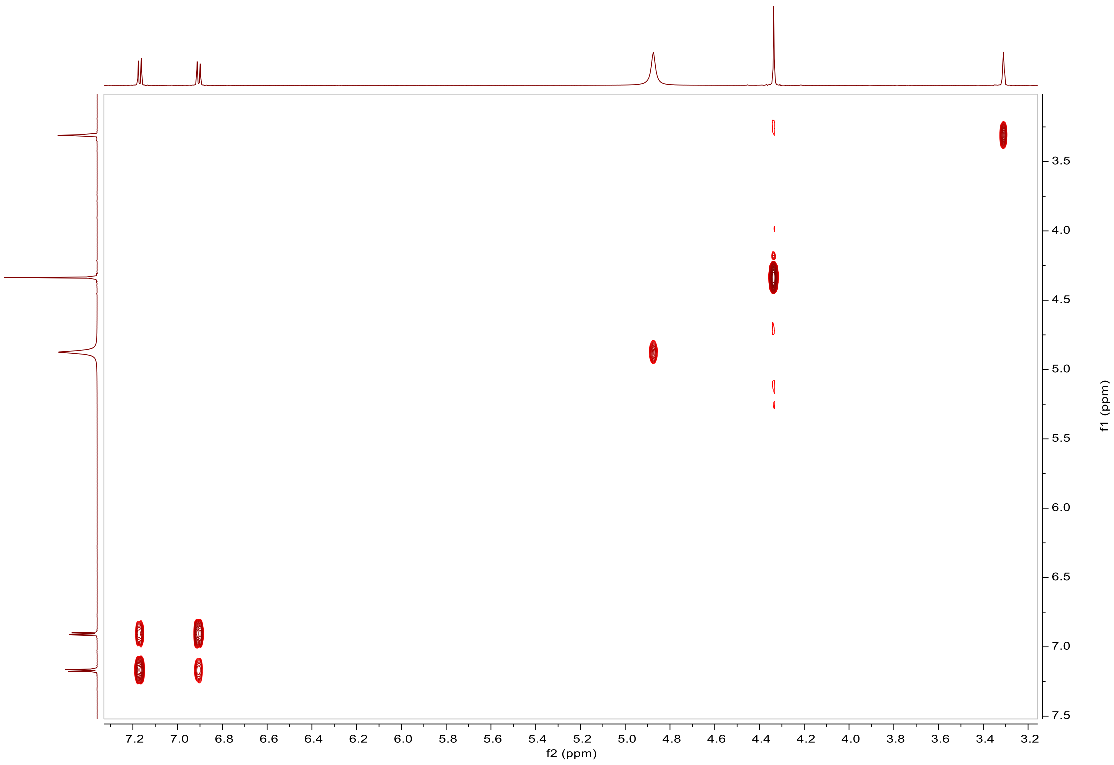

Figure S10. COSY spectrum of **2** in CD_3OD.

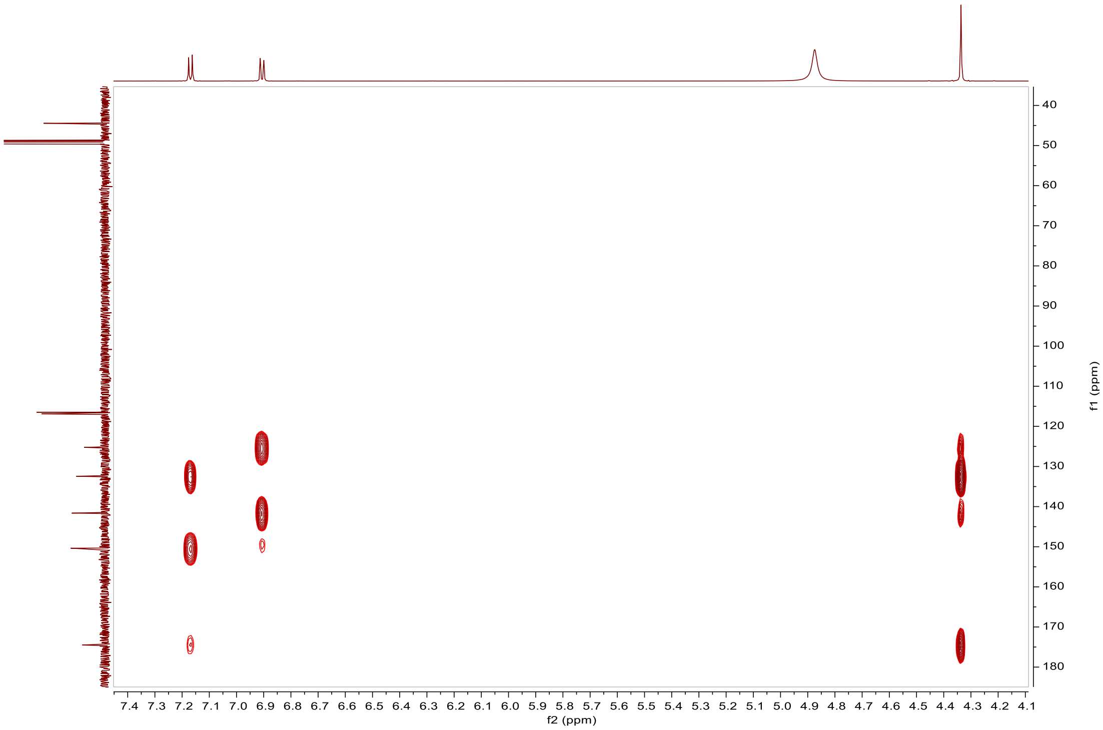

Figure S11. HMBC spectrum of **2** in CD_3OD.

Sample : 02. 2308-M-A2

(+) ESI-MS

Range : 100-250 m/z

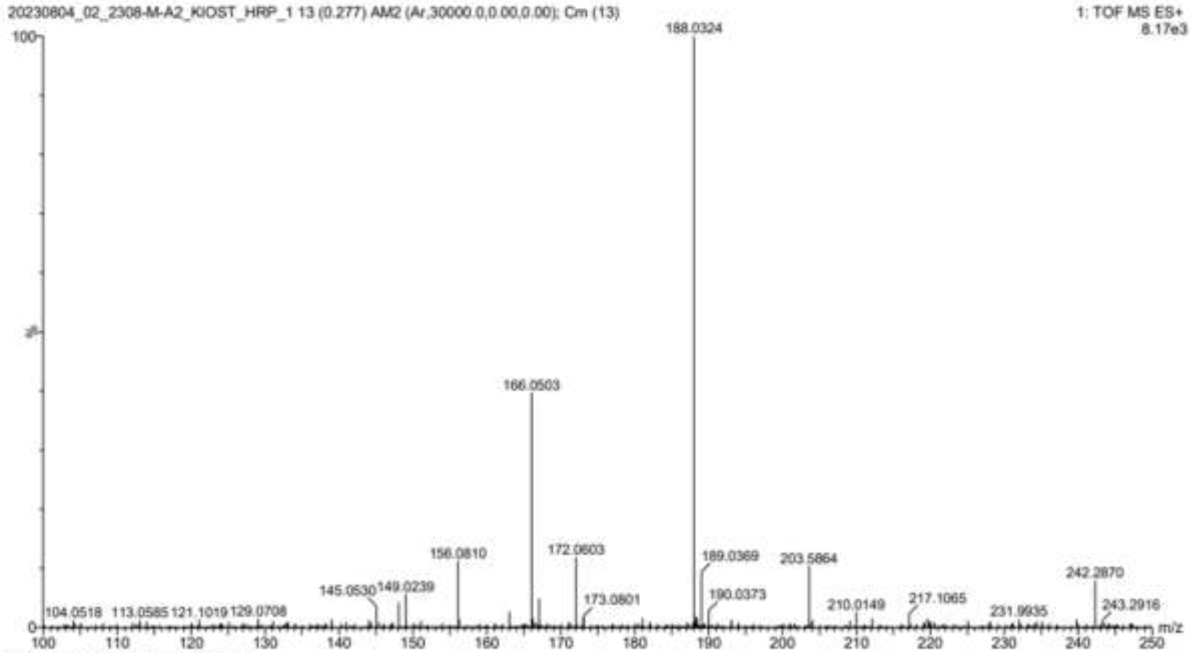

Elemental Composition Report

Single Mass Analysis
Tolerance = 10.0 PPM / DBE: min = -1.5, max = 50.0
Element prediction: Off
Number of isotope peaks used for i-FIT = 3

Monoisotopic Mass, Even Electron Ions
Elements Used:
C: 0-10 H: 0-300 N: 0-2 O: 0-5 Na: 0-1 Pt: 0-1

Minimum:				-1.5				
Maximum:		5.0	10.0	50.0				
Mass	Calc. Mass	mDa	PPM	DBE	i-FIT	Norm	Conf(%)	Formula
166.0503	166.0504	-0.1	-0.6	5.5	227.9	n/a	n/a	C8 H8 N O3
188.0324	188.0324	0.0	0.0	5.5	337.1	n/a	n/a	C8 H7 N O3 Na

Figure S12. HR-ESIMS spectrum of **2**.

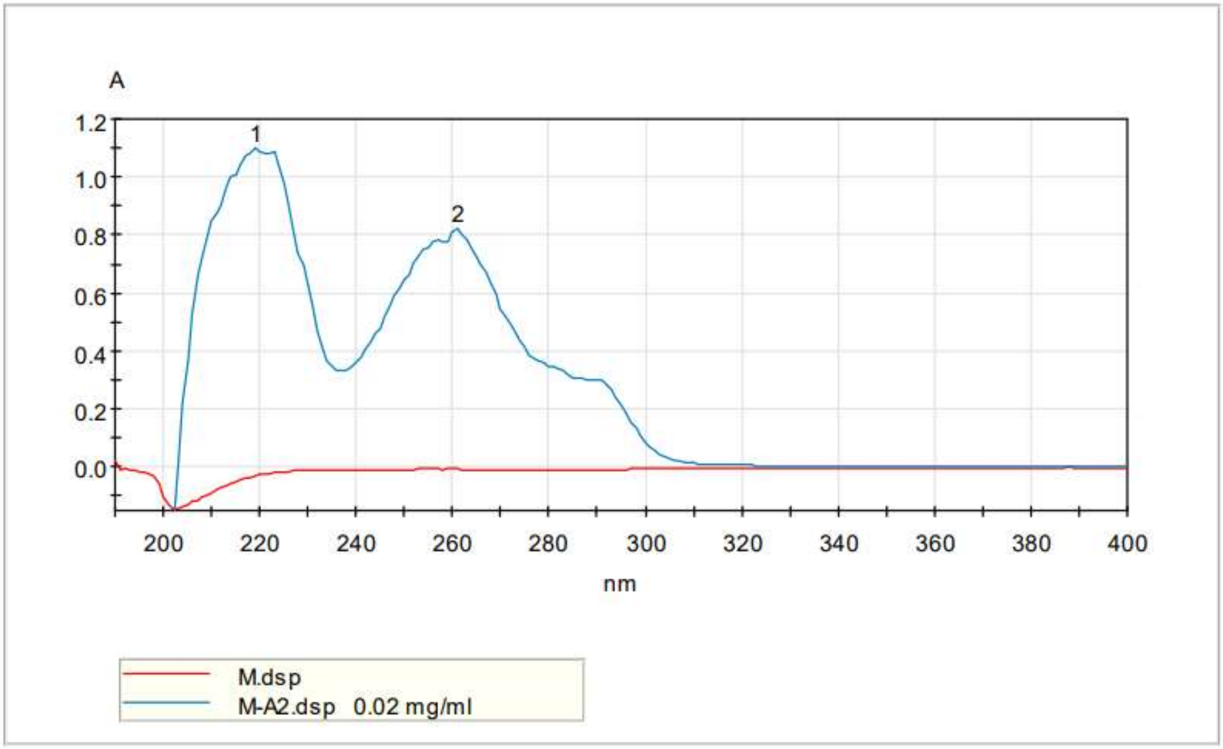

M-A2.dsp 0.02 mg/ml

**Maxima**		**Threshold: 0.1 A**				
**1**	**219 nm;**	**1.100 A**	**2**	**261 nm;**	**0.824 A**	

M-A2.dsp 0.02 mg/ml
290 nm 0.301 A

Figure S13. UV spectrum of **2**.

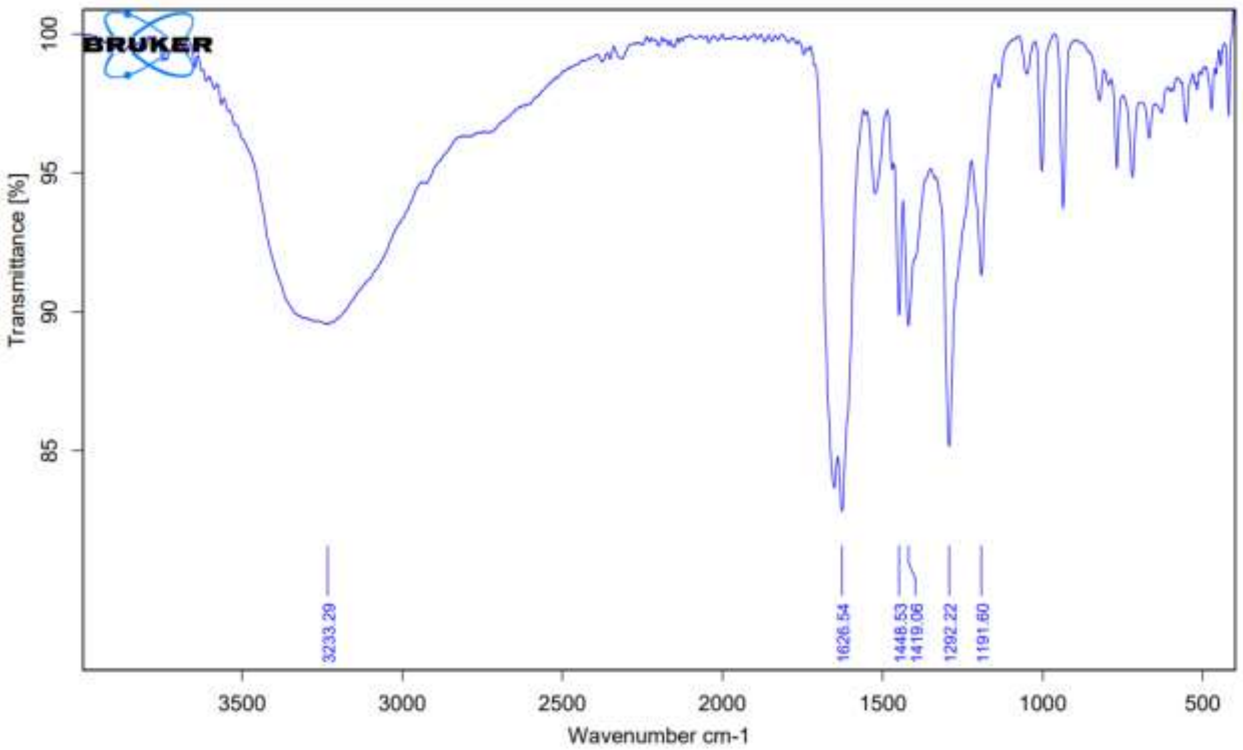

Figure S14. IR spectrum of **2**.

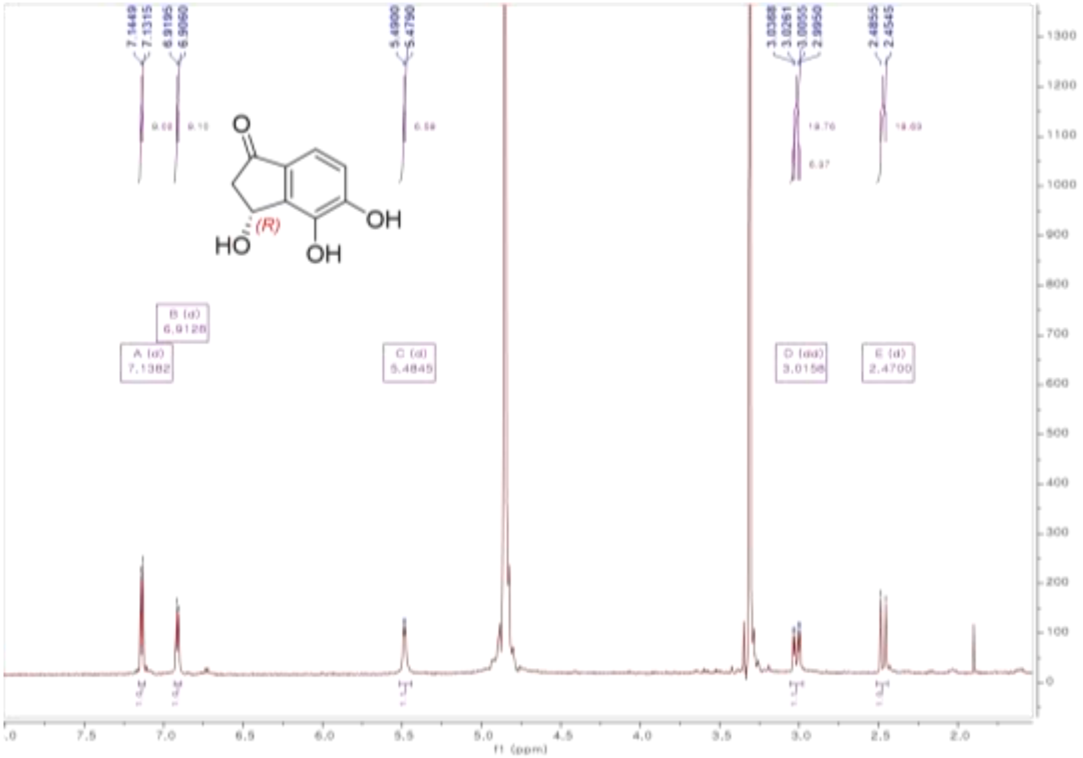

Figure S15. ^{1}H NMR spectrum of **3** in CD_3OD (600 MHz).

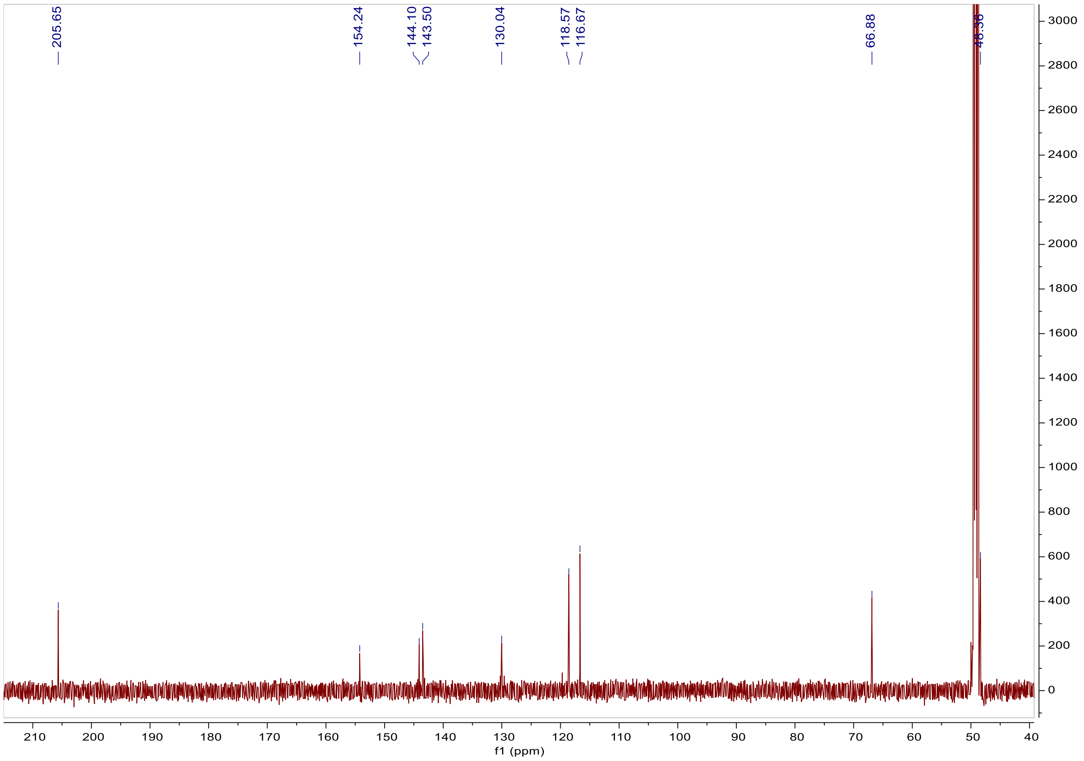

Figure S16. ^{13}C NMR spectrum of **3** in CD_3OD (150 MHz).

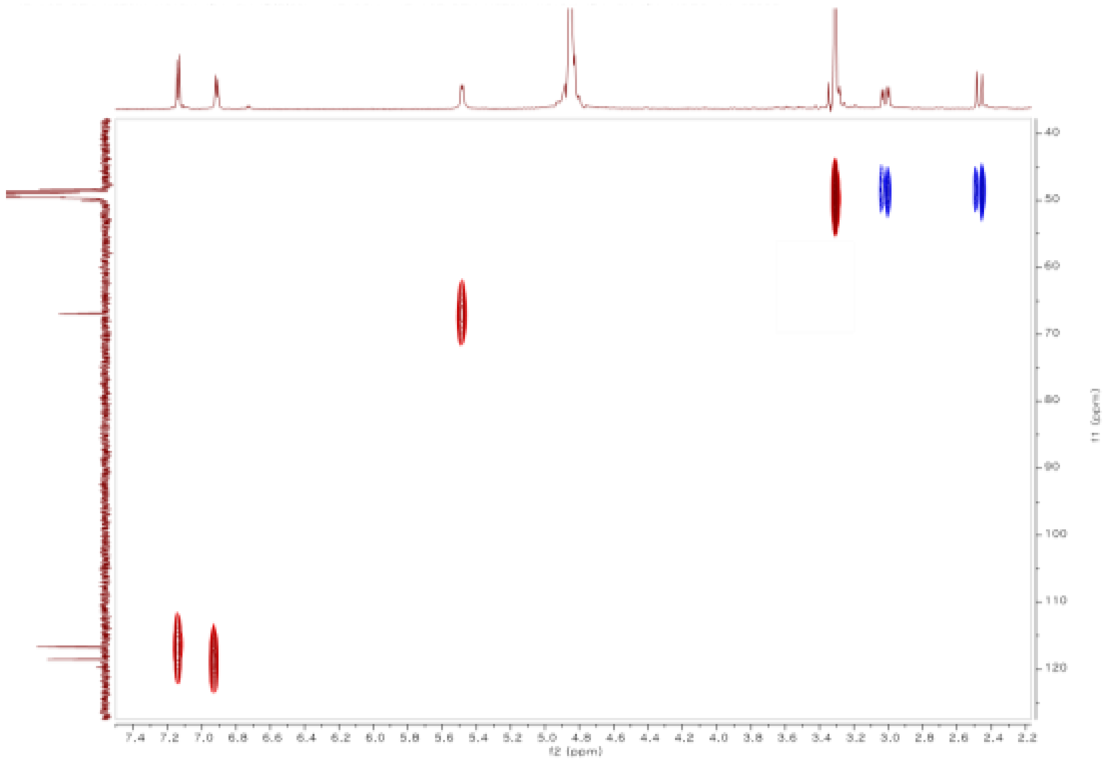

Figure S17. HSQC spectrum of **3** in CD_3OD.

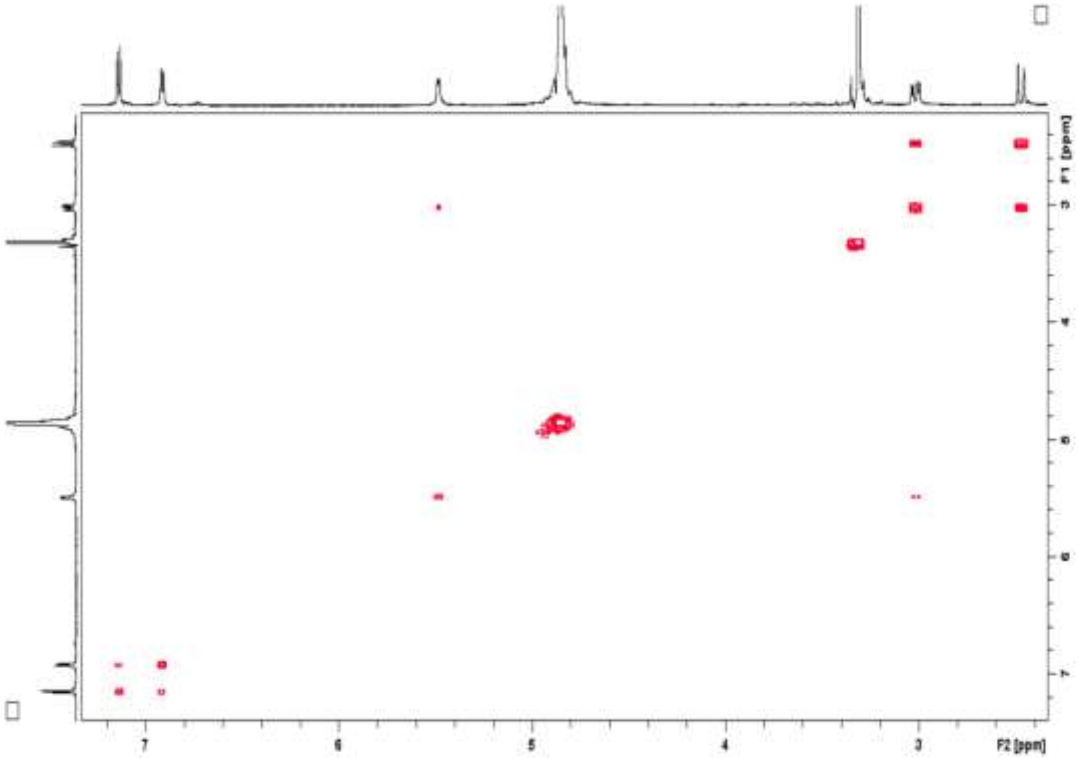

Figure S18. COSY spectrum of **3** in CD_3OD.

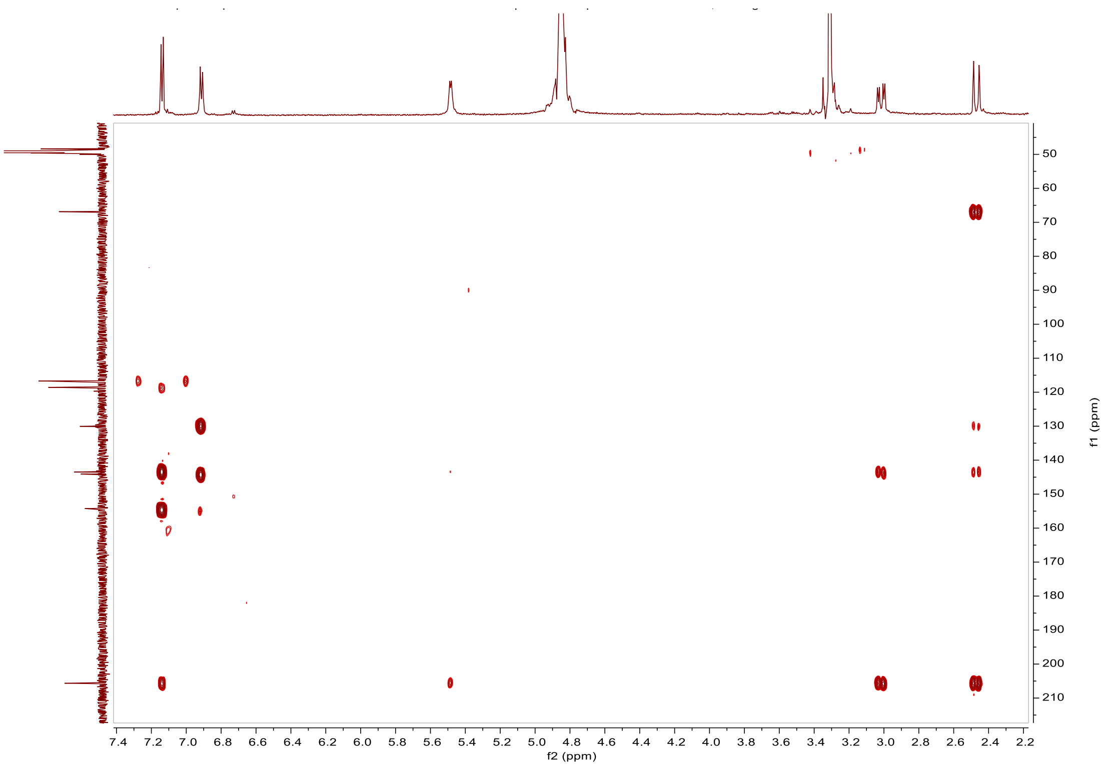

Figure S19. HMBC spectrum of **3** in CD_3OD.

Sample : 03. 2308-M-A3

(+) ESI-MS

Range : 150-250 m/z

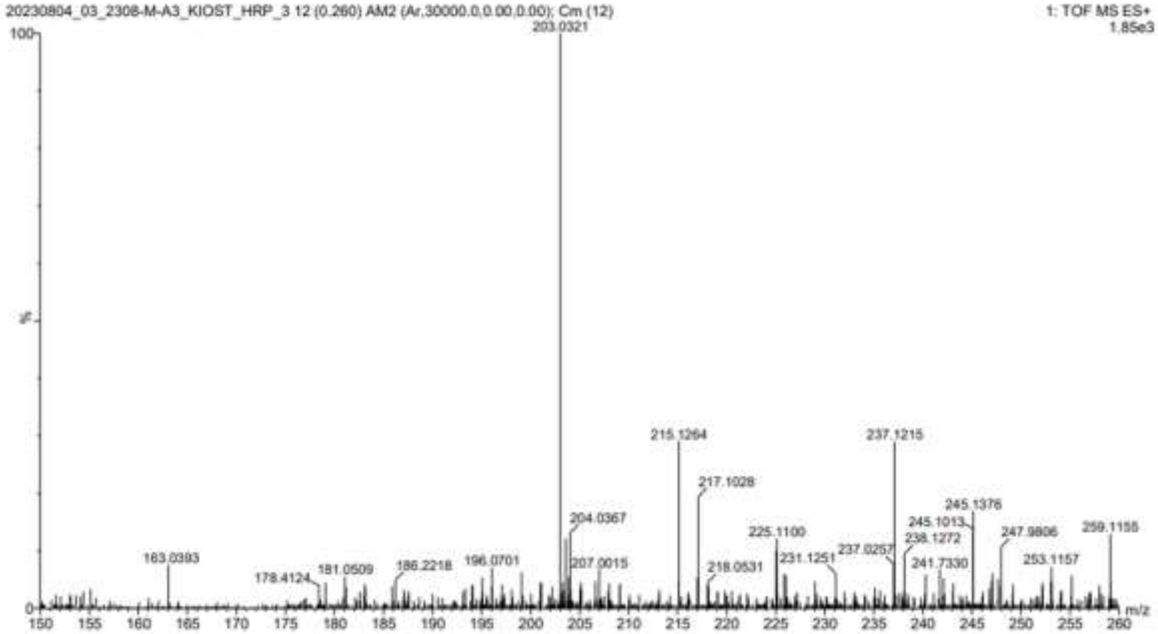

Elemental Composition Report

Single Mass Analysis
Tolerance = 10.0 PPM / DBE: min = -1.5, max = 50.0
Element prediction: Off
Number of isotope peaks used for i-FIT = 3

Monoisotopic Mass, Even Electron Ions
61 formula(e) evaluated with 1 results within limits (up to 50 closest results for each mass)
Elements Used:
C: 0-10 H: 0-300 N: 0-2 O: 0-5 Na: 0-1 Pt: 0-1

Minimum:				-1.5				
Maximum:		5.0	10.0	50.0				
Mass	Calc. Mass	mDa	PPM	DBE	i-FIT	Norm	Conf(%)	Formula
203.0321	203.0320	0.1	0.5	5.5	231.7	n/a	n/a	C9 H8 O4 Na

Figure S20. HR-ESIMS spectrum of **3**.

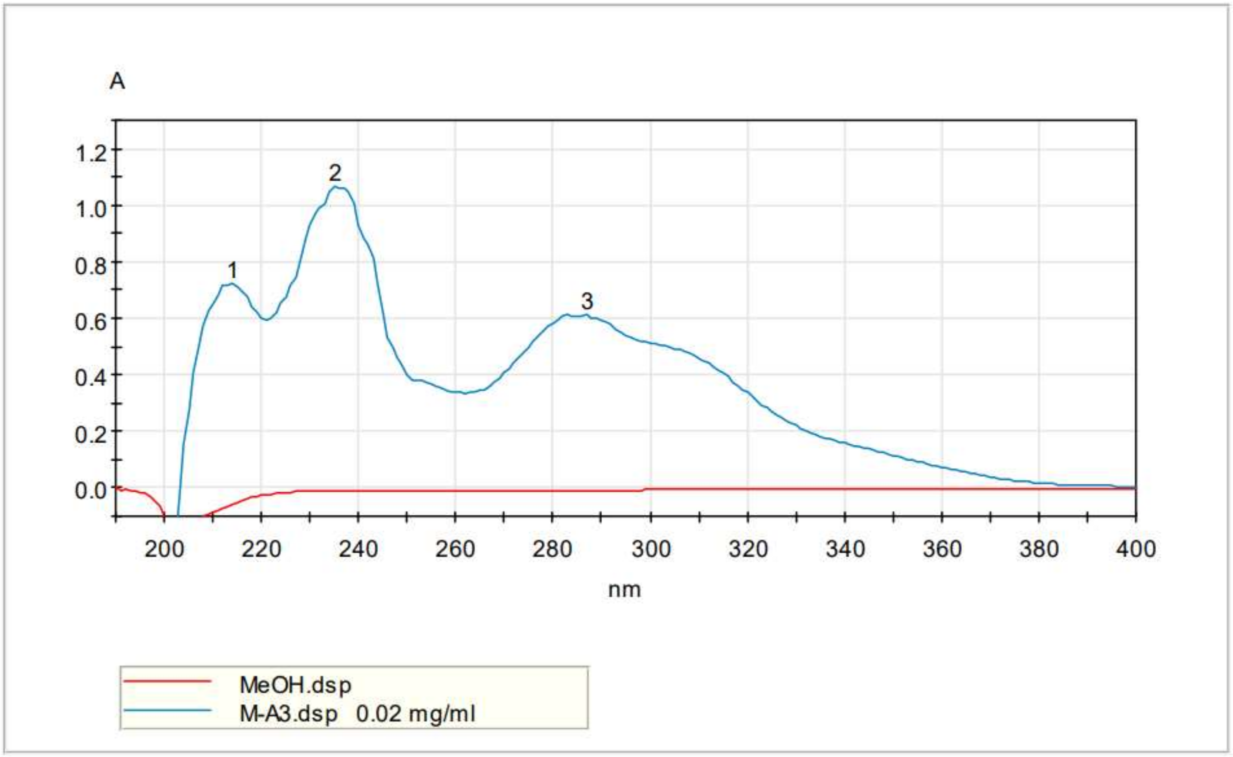

M-A3.dsp 0.02 mg/ml

Maxima	Threshold: 0.1 A							
1	214 nm;	0.725 A	2	235 nm;	1.068 A	3	287 nm;	0.613 A

Figure S21. UV spectrum of **3**.

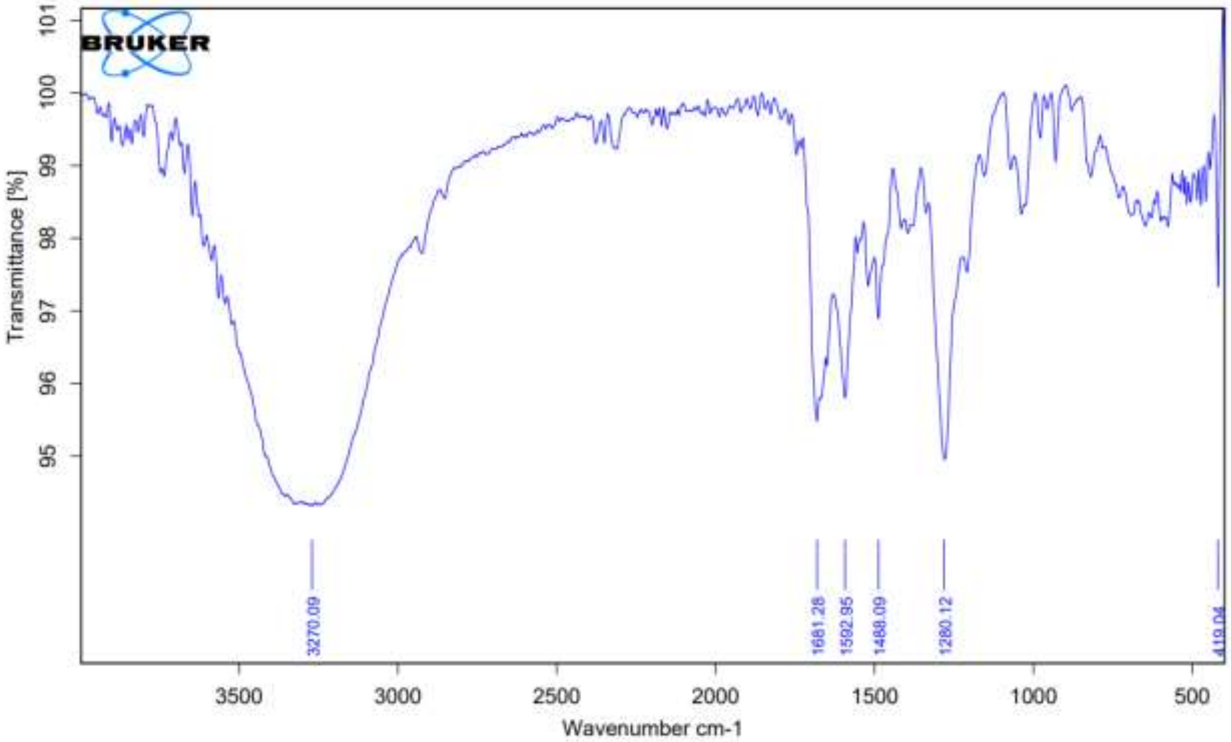

Figure S22. IR spectrum of **3**.

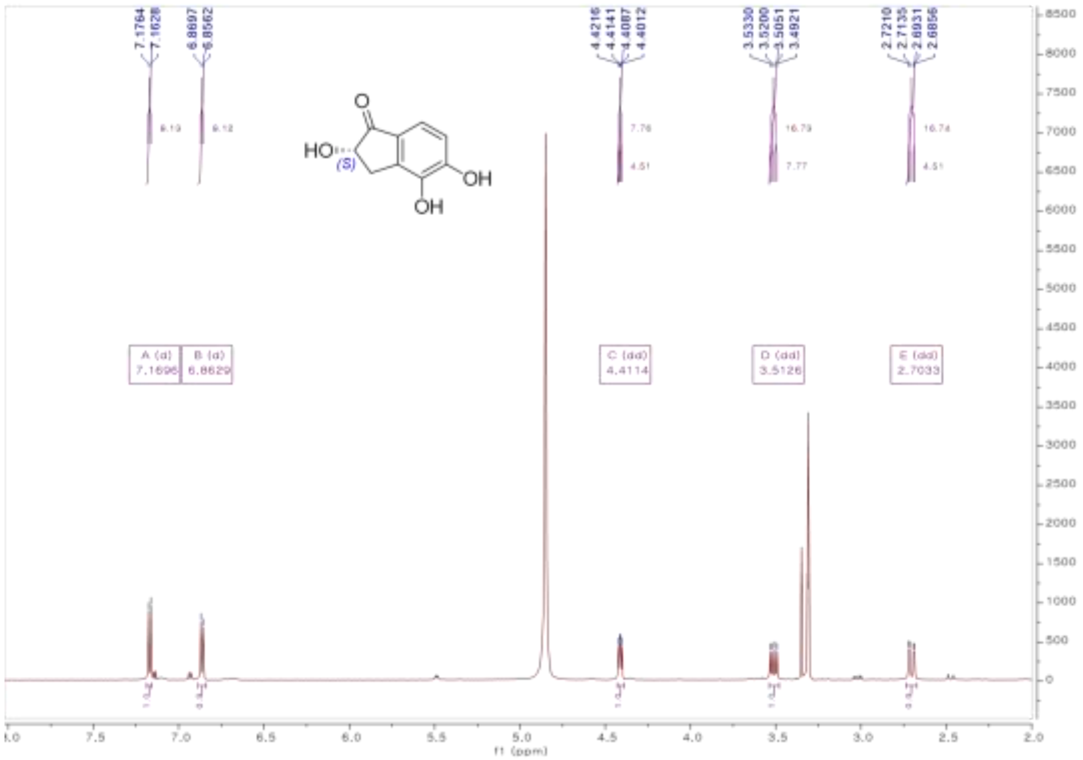

Figure S23. ^{1}H NMR spectrum of **4** in CD_3OD (600 MHz).

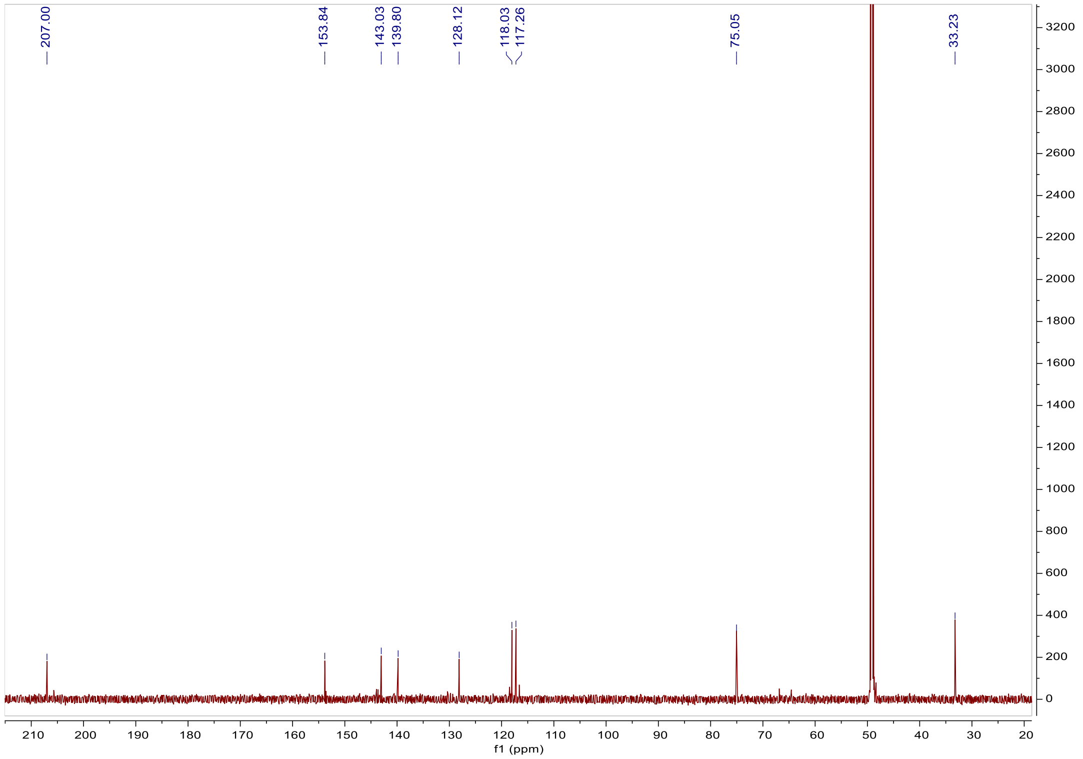

Figure S24. ^{13}C NMR spectrum of **4** in CD_3OD (150 MHz).

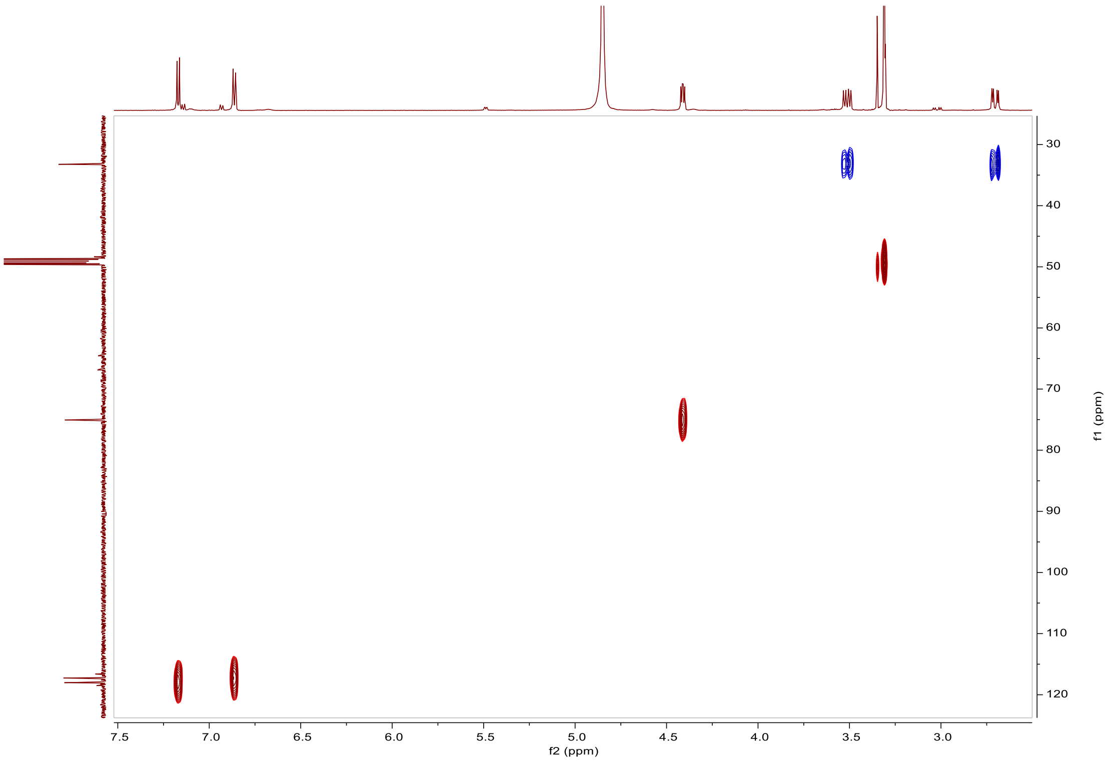

Figure S25. HSQC spectrum of **4** in CD_3OD.

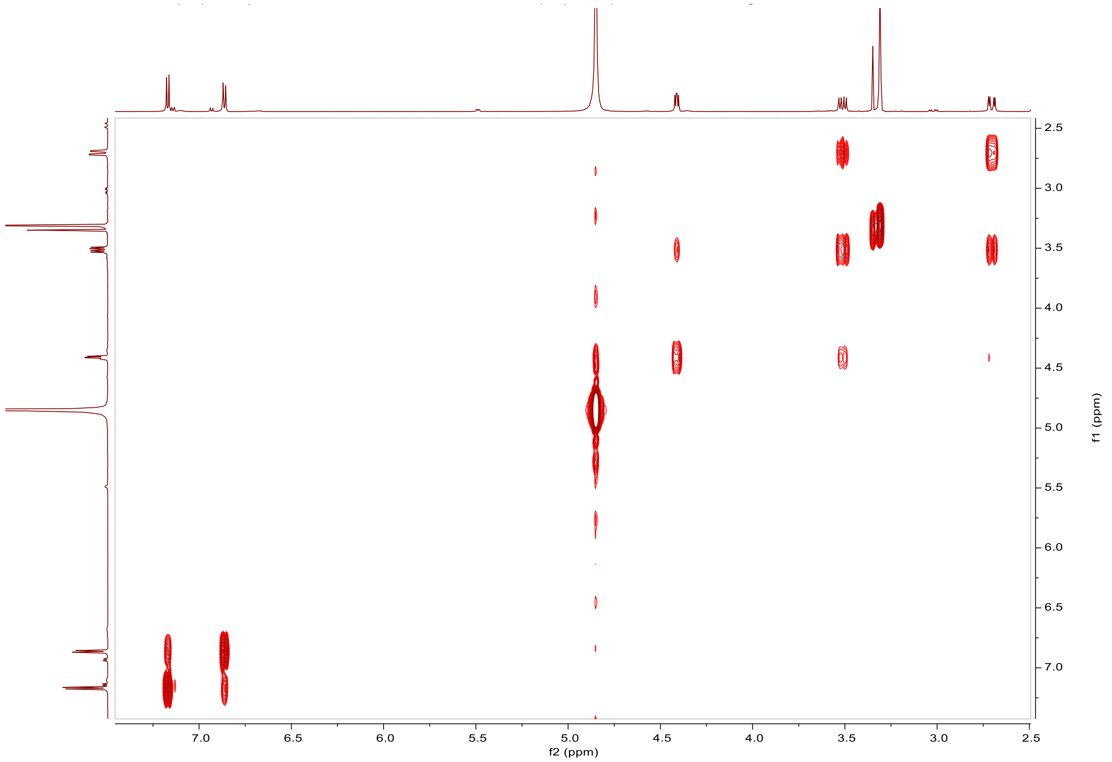

Figure S26. COSY spectrum of **4** in CD_3OD.

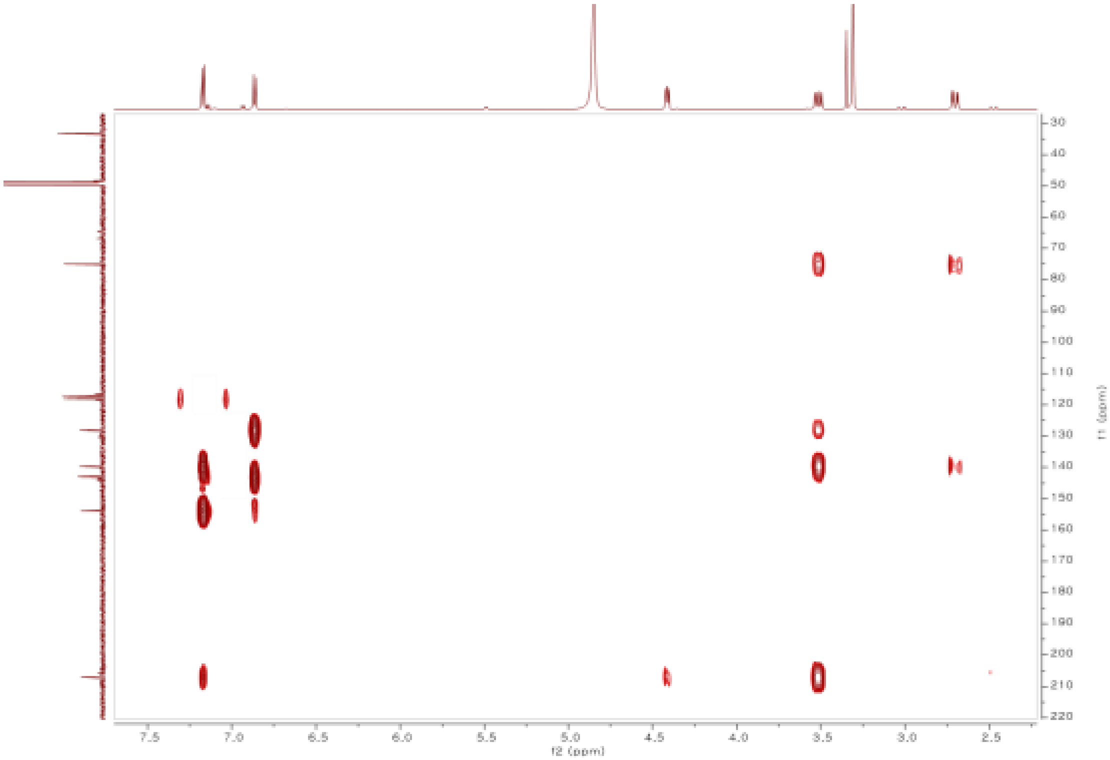

Figure S27. HMBC spectrum of **4** in CD_3OD.

Sample : 04. 2308-M-A4

(+) ESI-MS

Range : 150-260 m/z

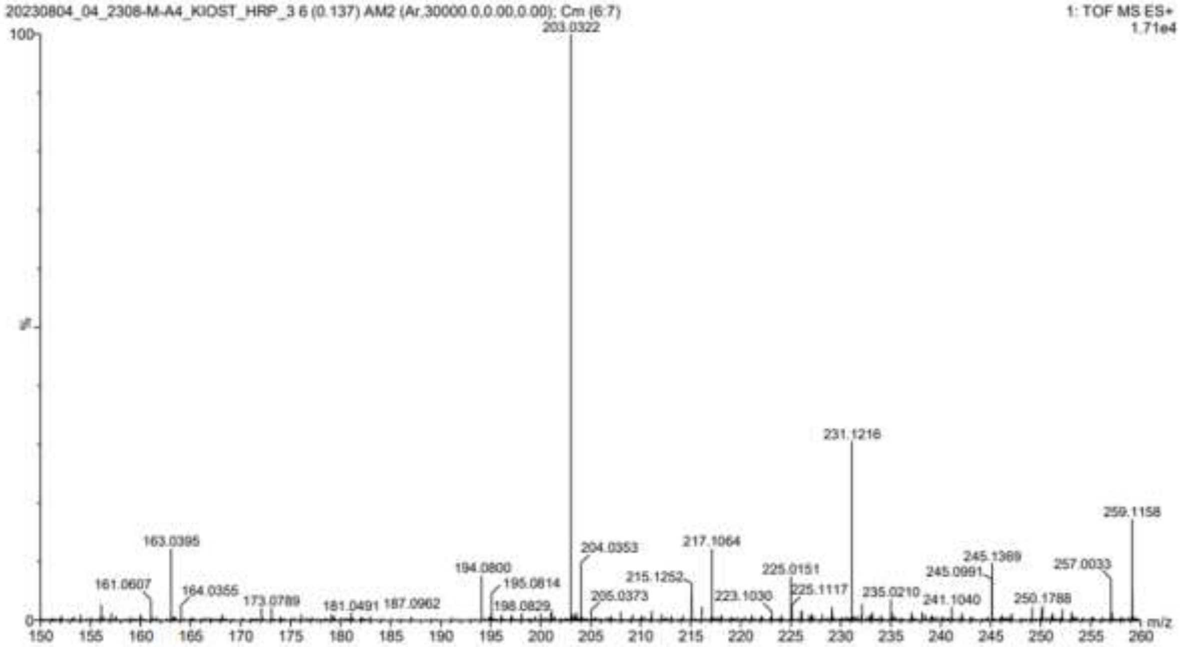

Elemental Composition Report

Single Mass Analysis
Tolerance = 10.0 PPM / DBE: min = -1.5, max = 50.0
Element prediction: Off
Number of isotope peaks used for i-FIT = 3

Monoisotopic Mass, Even Electron Ions
61 formula(e) evaluated with 1 results within limits (up to 50 closest results for each mass)
Elements Used:
C: 0-10 H: 0-300 N: 0-2 O: 0-5 Na: 0-1 Pt: 0-1

Minimum:				-1.5				
Maximum:		5.0	10.0	50.0				
Mass	Calc. Mass	mDa	PPM	DBE	i-FIT	Norm	Conf(%)	Formula
203.0322	203.0320	0.2	1.0	5.5	652.1	n/a	n/a	C9 H8 O4 Na

Figure S28. HR-ESIMS spectrum of **4**.

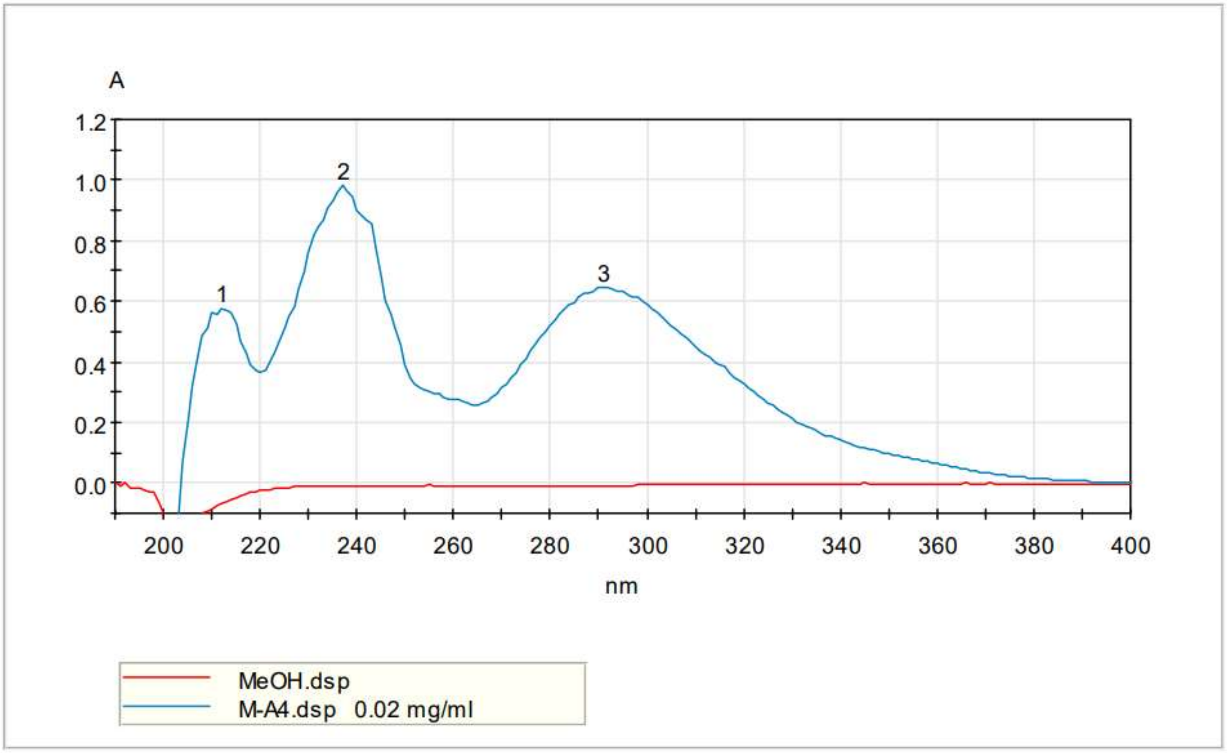

M-A4.dsp 0.02 mg/ml

Maxima	Threshold: 0.1 A							
1	212 nm;	0.578 A	2	237 nm;	0.983 A	3	291 nm;	0.647 A

Figure S29. UV spectrum of **4**.

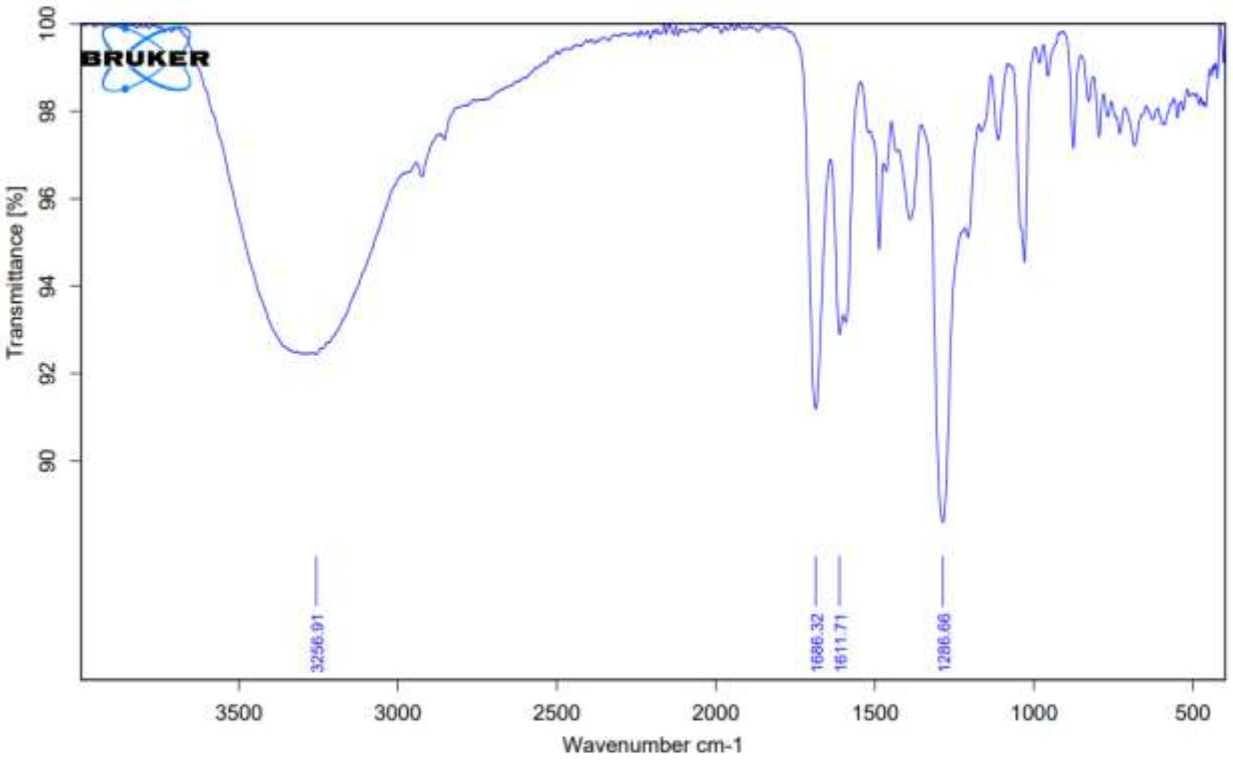

Figure S30. IR spectrum of **4**.

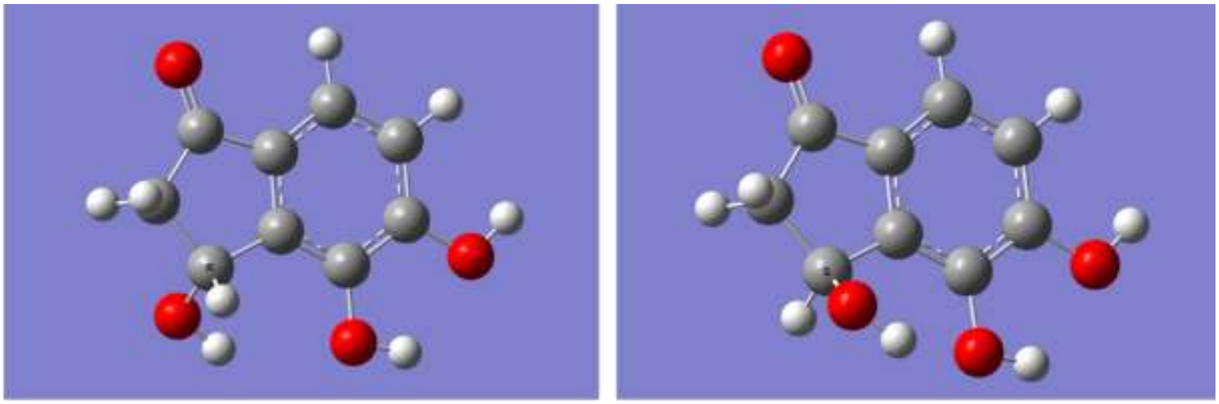

Conformer 1 of **3**-3*R* Conformer 1 of **3**-3*S*

Figure S31. Initial geometry optimized conformers of **3**.

Table S1. Total Gibbs Free Energy and Boltzmann population of initial geometry optimized conformers of **3**.

Compound	Conformer	Total Gibbs Free Energy (KCAL/MOL)	Boltzmann population (%)
**3**-3*R*	1	86.85337	99.98721
**3**-3*S*	1	86.85337	99.98721

Table S2. The cartesian coordinates of conformers of **3**.

		Coordinates (Angstroms)		
		X	Y	Z
	C	-0.13935	0.35933	-0.15283
	C	1.22404	0.58074	-0.12869
	C	2.07715	-0.51859	0.02033
	C	1.56865	-1.81474	0.14007
	C	0.19733	-2.03407	0.11963
	C	-0.64369	-0.93326	-0.02294
	H	2.2602	-2.64382	0.25386
	H	-0.20618	-3.0356	0.21872
	C	-2.52303	0.59145	-0.11556
	C	-2.11533	-0.87483	-0.04662
Conformer 1 of **3**-3*R*	O	-2.88715	-1.81987	-0.01033
	O	1.70662	1.85003	-0.24188
	O	3.40294	-0.2145	0.03601
	H	2.6748	1.83643	-0.19793
	H	3.95222	-1.0034	0.13717
	H	-3.266	0.74618	-0.90068
	H	-2.98227	0.87118	0.83818
	C	-1.23005	1.39211	-0.3165
	H	-1.18955	1.82497	-1.32352
	O	-1.15192	2.43797	0.64824
	H	-0.30135	2.88635	0.5365

		Coordinates (Angstroms)		
		X	Y	Z
	C	0.13934	0.35935	-0.15269
	C	-1.22404	0.58079	-0.12858
	C	-2.0772	-0.51851	0.02041
	C	-1.56873	-1.81469	0.14013
	C	-0.19741	-2.03405	0.11972
	C	0.64364	-0.93326	-0.02279
	H	-2.26034	-2.64373	0.25381
	H	0.20607	-3.03559	0.21876
	C	2.52305	0.59133	-0.11576
	C	2.11527	-0.8749	-0.04665
Conformer 1 of **3**-3*S*	O	2.88705	-1.81998	-0.01036
	O	-1.70654	1.8501	-0.2417
	O	-3.40294	-0.21443	0.0358
	H	-2.67475	1.83646	-0.19865
	H	-3.95227	-1.00332	0.1367
	H	3.26587	0.74596	-0.90105
	H	2.98247	0.87115	0.83786
	C	1.23007	1.39208	-0.3165
	H	1.18941	1.8249	-1.32354
	O	1.15219	2.43795	0.64822
	H	0.30157	2.88633	0.53672

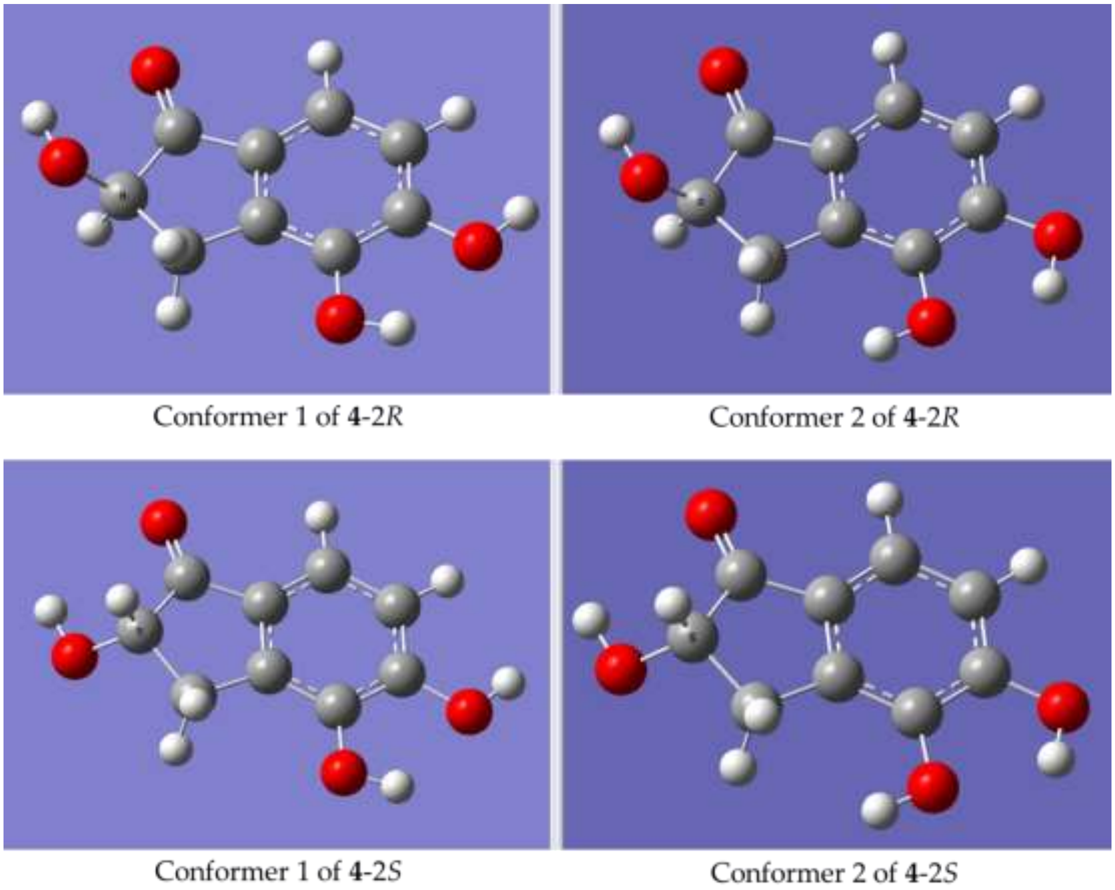

Figure S32. Initial geometry optimized conformers of **4**.

Table S3. Total Gibbs Free Energy and Boltzmann population of initial geometry optimized conformers of **4**.

Compound	Conformers	Total Gibbs Free Energy (KCAL/MOL)	Boltzmann population (%)
4-2*R*	1	119.3421	83.75961
	2	120.2777	16.14874
4-2*S*	1	119.3421	83.75961
	2	120.2777	16.14874

Table S4. The cartesian coordinates of conformers of **4**.

		Coordinates (Angstroms)		
		X	Y	Z
Conformer 1 of **4**-2*R*	C	-0.06733	-0.51437	0.04284
	C	-1.42593	-0.76952	0.00497
	C	-2.3109	0.3163	-0.02761
	C	-1.85078	1.63557	-0.02646
	C	-0.48834	1.89374	0.00949
	C	0.38668	0.81046	0.05192
	H	-2.57061	2.44789	-0.051
	H	-0.11499	2.91178	0.0144
	C	2.28939	-0.64388	0.37068
	C	1.84886	0.7967	0.09485
	O	2.63487	1.71834	-0.0626
	O	3.42835	-1.00697	-0.37827
	H	4.05832	-0.27227	-0.32996
	O	-1.88836	-2.04924	-0.01138
	O	-3.62656	-0.02718	-0.06452
	H	-2.85715	-2.03781	-0.03804
	H	-4.2009	0.74925	-0.10249
	H	2.50444	-0.70149	1.44869
	C	1.06673	-1.50829	0.04336
	H	0.91312	-2.32073	0.75668
	H	1.19107	-1.95649	-0.94826
Conformer 2 of **4**-2*R*	C	-0.07855	-0.509	0.04886
	C	-1.43978	-0.74557	0.0056
	C	-2.32037	0.34504	-0.02926
	C	-1.84608	1.65901	-0.02737
	C	-0.48249	1.90336	0.00939
	C	0.38567	0.81404	0.05435
	H	-2.56723	2.46816	-0.05611
	H	-0.09809	2.9174	0.01233
	C	2.28301	-0.64686	0.37229
	C	1.84564	0.79422	0.09605
	O	2.63639	1.71192	-0.06279
	O	3.41081	-1.01801	-0.38898
	H	4.04289	-0.28422	-0.35405
	O	-2.03823	-1.97503	-0.01278
	O	-3.65568	0.12767	-0.07231
	H	-1.39376	-2.69297	-0.0547
	H	-3.82526	-0.82755	-0.08087
	H	2.50932	-0.70431	1.44763
	C	1.0548	-1.50898	0.05723
	H	0.91406	-2.31106	0.78661
	H	1.18072	-1.96935	-0.92875

		Coordinates (Angstroms)		
		X	Y	Z
Conformer 1 of **4**-2*S*	C	0.06733	-0.51437	0.04284
	C	1.42593	-0.76952	0.00497
	C	2.3109	0.3163	-0.02761
	C	1.85078	1.63557	-0.02646
	C	0.48834	1.89374	0.00949
	C	-0.38668	0.81046	0.05192
	H	2.57061	2.44789	-0.051
	H	0.11499	2.91178	0.0144
	C	-2.28939	-0.64388	0.37068
	C	-1.84886	0.7967	0.09485
	O	-2.63487	1.71834	-0.0626
	O	1.88836	-2.04924	-0.01138
	O	3.62656	-0.02718	-0.06452
	H	2.85715	-2.03781	-0.03804
	H	4.2009	0.74925	-0.10249
	O	-3.42835	-1.00697	-0.37827
	H	-4.05832	-0.27227	-0.32996
	H	-2.50444	-0.70149	1.44869
	C	-1.06673	-1.50829	0.04336
	H	-1.19107	-1.95649	-0.94826
	H	-0.91312	-2.32073	0.75668
Conformer 2 of **4**-2*S*	C	0.07855	-0.509	0.04886
	C	1.43978	-0.74557	0.0056
	C	2.32037	0.34504	-0.02926
	C	1.84608	1.65901	-0.02737
	C	0.48249	1.90336	0.00939
	C	-0.38567	0.81404	0.05435
	H	2.56723	2.46816	-0.05611
	H	0.09809	2.9174	0.01233
	C	-2.28301	-0.64686	0.37229
	C	-1.84564	0.79422	0.09605
	O	-2.63639	1.71192	-0.06279
	O	2.03823	-1.97503	-0.01278
	O	3.65568	0.12767	-0.07231
	H	1.39376	-2.69297	-0.0547
	H	3.82526	-0.82755	-0.08087
	O	-3.41081	-1.01801	-0.38898
	H	-4.04289	-0.28422	-0.35405
	H	-2.50932	-0.70431	1.44763
	C	-1.0548	-1.50898	0.05723
	H	-1.18072	-1.96935	-0.92875
	H	-0.91406	-2.31106	0.78661
